# Integrating network pharmacology, IPA, and molecular docking to reveal the anti-osteoporosis effects of EA and EB via the FAK pathway

**DOI:** 10.3389/fphar.2025.1532665

**Published:** 2025-07-02

**Authors:** Ziheng Wei, Fei You, Henghui Li, Si Wu, Fen Tang, Xiangyang Wan, Huizhong Dong, Wenxuan Huang, Songyan Gao, Bo Cai, Xiongsheng Chen, Xin Dong

**Affiliations:** ^1^ School of Medicine, Shanghai Jiao Tong University, Shanghai, China; ^2^ Department of Orthopaedics, Shanghai General Hospital, Shanghai, China; ^3^ School of Medicine, Shanghai University, Shanghai, China; ^4^ Departments of Genetics, School of Medicine, Stanford University, Stanford, CA, United States; ^5^ Department of School Health, Shanghai Baoshan Center for Disease Control and Prevention, Shanghai, China

**Keywords:** osteoporosis, anti-osteoporosis drugs, epimedium, network pharmacology, molecular docking, IPA

## Abstract

Osteoporosis is a widespread condition among the elderly, with a particularly high incidence in postmenopausal women aged 50 and above. This disease significantly increases the risk of fractures, adversely affecting the quality of life. *Epimedium*, a traditional Chinese medicinal herb, has been widely used in the treatment of osteoporosis due to its diverse therapeutic properties. However, *Epimedium* contains a complex mixture of compounds, including both beneficial and potentially harmful constituents. Therefore, there is a critical need to identify and isolate active monomeric compounds that can effectively treat osteoporosis, thereby enhancing the specificity and efficacy of treatment while reducing the intake of harmful substances. Through an integrated approach utilizing network pharmacology and extensive literature review, we identified five previously unreported anti-osteoporotic monomeric compounds from various traditional agents: Epimedin A (EA), Epimedin B (EB), Epimedoside A (EPA), 4-Hydroxybenzaldehyde (PHBA), and Baohuoside VI. We subsequently evaluated the effects of these compounds on bone marrow-derived macrophages (BMMs) and cranial preosteoblasts. Results from tartrate-resistant acid phosphatase (TRAP) staining and quantitative polymerase chain reaction (qPCR) demonstrated that EA, EB, and EPA significantly inhibited BMM differentiation into osteoclasts in a dose-dependent manner. In contrast, alkaline phosphatase (ALP) staining, Alizarin Red staining, and qPCR results showed that EA and EB promoted the differentiation of cranial preosteoblasts into osteoblasts in a dose-dependent fashion. Furthermore, intraperitoneal administration of EA and EB at doses of 5 mg/kg, 10 mg/kg, and 20 mg/kg in ovariectomized (OVX) mice resulted in a significant increase in bone mineral density and trabecular bone number compared to the OVX group (P < 0.05 compared to OVX group). These findings suggest that EA and EB may mitigate bone loss in OVX mice. Importantly, high doses of EA and EB did not exhibit pharmacological toxicity in various organs, as confirmed by hematoxylin and eosin (HE) staining. In exploring the underlying mechanisms, we found that EA and EB do not modulate the NF-κB signaling pathway, as indicated by the NFKB luciferase reporter assay. Western blot analysis further revealed that EA and EB might not affect osteoporosis progression via the MAPK (ERK and JNK) or NF-κB (P65 and IκBα) pathways. To elucidate the molecular targets, we utilized PharmMapper, Similarity Ensemble Approach, SwissTargetPrediction, and SuperPred to predict potential targets of EA and EB. Intersection analysis using the Ingenuity Pathway Analysis (IPA) database indicated that EA and EB regulate the focal adhesion kinase (FAK) signaling pathway. Molecular docking studies using Autodock confirmed the binding of EA and EB to FAK1 (binding free energy: −13.012 kJ/mol and −14.0164 kJ/mol) and FAK2 (binding free energy: −5.815 kJ/mol and −6.4852 kJ/mol). qPCR analysis further demonstrated that EA and EB significantly inhibited FAK1 and FAK2 gene expression in osteoclasts while promoting their expression in osteoblasts at very high doses. In conclusion, EA and EB, identified as active monomeric compounds in *Epimedium*, may exert their anti-osteoporotic effects by modulating the FAK signaling pathway, thereby enhancing bone mineral density and improving the quality of life for patients with osteoporosis. This study provides new insights into the pathogenesis of osteoporosis and the development of targeted anti-osteoporosis therapies. Further research is warranted to validate the role of EA and EB in modulating osteoporosis progression via the FAK signaling pathway.

## Introduction

Osteoporosis is a metabolic skeletal disorder characterized by reduced bone mass and the deterioration of bone tissue microarchitecture, leading to increased susceptibility to fractures (Gopinath, 2023). These osteoporotic fractures, particularly hip fractures, are associated with significant morbidity and mortality, placing a substantial financial burden on healthcare systems. Individuals who suffer from osteoporosis-related fractures have a higher likelihood of experiencing subsequent fractures, which further exacerbates morbidity and increases the risk of premature mortality (Briot, 2017; van Geel et al., 2009; van Geel et al., 2010). In the United States alone, osteoporosis is responsible for more than 2 million fractures each year (Author anonymous, 2021). Globally, approximately one in three women and one in five men over the age of 50 will experience an osteoporotic fracture in their lifetime (Yu and Xia, 2019). Although age-adjusted rates of fragility fractures are decreasing, the absolute number of such fractures is rising, largely due to the growing population of older adults (Friedman and Mendelson, 2014). Among all fragility fractures, hip fractures are associated with the highest rates of disability and mortality, with over 300,000 cases reported annually (Author anonymous, 2021). Following a hip fracture, mortality rates range from 20% to 30% within the first year (Curry et al., 2018). Surgical intervention for hip fractures carries a 4% mortality risk, and the first 3 months post-surgery are associated with a 5 to 8-fold increase in all-cause mortality (Friedman and Mendelson, 2014). Hospitalization is almost always required for hip fractures, with up to 25% of patients needing long-term care and 50% experiencing a lasting loss of mobility (Author anonymous, 2021).

Recent advances in osteoporosis treatment have introduced contemporary therapeutic options, including newer anabolic agents such as teriparatide, abaloparatide, and romosozumab, which have shown promise in managing severe osteoporosis (Langdahl, 2021). However, pharmacological treatment of osteoporosis presents several challenges. Many medications require long-term or even lifelong adherence, which increases the risk of various complications, including rare but serious side effects like atypical femur fractures and osteonecrosis of the jaw (Gedmintas et al., 2013; Khan et al., 2015). Consequently, there is a pressing need to discover new drugs that are not only highly effective but also have minimal side effects for the treatment of osteoporosis.

Traditional Chinese Medicine (TCM) has garnered increasing attention for its efficacy in treating various diseases with fewer side effects. Recent scientific literature indicates that TCM interventions can have both anabolic and anticatabolic effects in osteoporosis treatment by promoting bone formation and regulating bone resorption (Suvarna et al., 2018), leading to improved bone mineral density, enhanced biomechanical properties, and the preservation of bone microstructure (Wang et al., 2019; Wang et al., 2017; Indran et al., 2016). The dried aerial parts of *Epimedium* have been traditionally used, either alone or in combination with other herbs, for centuries to enhance bone health (Wang et al., 2019). Despite numerous *in vitro* and *in vivo* studies confirming the efficacy of *Epimedium* in osteoporosis management, the specific active ingredients and underlying mechanisms of action remain to be fully elucidated.

Network pharmacology, based on the “multi-compound, multi-target” paradigm, aligns well with the holistic principles of TCM and provides a suitable methodology for comprehensively investigating the molecular mechanisms underlying the effects of *Epimedium* (Chen et al., 2014). Previous studies conducted by our research team have demonstrated the effectiveness of network pharmacology in clarifying the mechanisms of action associated with TCM (Chen et al., 2014; Tang et al., 2022; Xing et al., 2018; Chen et al., 2016). In the present study, we employed network pharmacology, Ingenuity Pathway Analysis (IPA), and molecular docking as computational tools to unravel the complex mechanisms through which *Epimedium* exerts its anti-osteoporotic effects, particularly through the modulation of FAK1 and FAK2 pathways. Subsequently, we conducted a series of pharmacological experiments to systematically explore the inhibitory effects of *Epimedium* on osteoclast and osteoblast activity, focusing on the potential role of the FAK signaling pathway, thereby validating the outcomes derived from our network pharmacology approach.

## Methods

### Ovariectomy-induced osteoporosis in a mouse model

Twenty-four female C57BL/6J mice (12 weeks old, weighing 16–18 g) were obtained from Shanghai Model Biology Center Co., Ltd. All animal procedures were approved by the Ethics Committee of Shanghai University (approval number: ECSHU 2021-168). The mice were housed in a specific pathogen-free (SPF) facility at the Institute of Translational Medicine, Shanghai University. Food, water, and bedding changes were managed by facility staff, ensuring that the mice had free access to food and water. All experiments were conducted in strict compliance with the guidelines of the Ethics Committee of Shanghai University (SYXK 2020-0033).

After 1 week of acclimation, the C57BL/6J female mice were randomly assigned to four groups (n = 6 per group): sham group, model group, and two experimental groups. All surgical instruments were autoclaved before use, and fat-free cotton balls and iodophor were prepared for the procedures. General anesthesia was induced via inhalation of 0.41 mL/min of isoflurane at 4L/min fresh gas flow. The fur on the backs of the mice was shaved using a razor to prepare for surgery. Bilateral ovariectomy was performed on mice in the model and experimental groups to induce bone loss and structural deterioration characteristic of osteoporosis. Sham-operated mice underwent the same surgical procedure without the removal of ovaries. Post-surgery, iodophor was applied to each mouse’s incision site to prevent infection.

Following surgery, the mice were given a 1-week recovery period before treatment commenced. Mice in the model and sham groups received intraperitoneal injections of normal saline (NS) every 2 days, whereas mice in the experimental groups were injected with EA or EB compounds at specified concentrations every 2 days for a total duration of 6 weeks. All mice were weighed weekly throughout the treatment period. At the end of the 6-week treatment, all mice were euthanized via cervical dislocation. The left femurs were harvested and fixed in 4% paraformaldehyde for 2 days to prepare them for histological evaluation and micro-computed tomography (micro-CT) analysis.

### Micro-CT scanning

Prior to decalcification, the intact left femur was scanned using a Micro-CT system (Bruker micro-CT, Kontich, Belgium). The procedure began by positioning the distal femur horizontally on the scanning stage, where it was stabilized and secured to ensure it remained intact within the field of view throughout the scanning process, facilitating subsequent sample replacement. The scanning parameters were set as follows: rotation step of 0.20°, pixel size of 9 μm, voltage of 70 kV, and current of 142 μA.

After scanning, the Region of Interest (ROI) within the distal femur was selected using Data-Viewer software, and the data were saved for analysis. CT-An software (Version 1.1) was then employed to analyze the acquired data. A threshold value of 65 was applied to extract bone-related parameters, including bone volume to tissue volume ratio (BV/TV), trabecular thickness (Tb.Th), trabecular number (Tb.N), connectivity density (Conn.Dn), bone surface area (BS), and trabecular separation (Tb.Sp). Finally, CT-Vol software (Version 1.14) was used to reconstruct the bone structure, providing clear global three-dimensional images of the distal femur.

### Histological analysis

Following micro-CT scanning, the fixed femur samples were decalcified in 10% EDTA for 2 weeks. The decalcified samples were then embedded in paraffin blocks and sectioned into 5 μm thick slices. These sections were stained with hematoxylin-eosin or tartrate-resistant acid phosphatase (TRAP) and observed under a light microscope (Nikon Corporation, Minato, Tokyo, Japan) at magnifications of ×40 and 100X.

### Osteoclasts extraction and culture

Female C57BL/6J mice (4–6 weeks old) were euthanized using cervical dislocation and subsequently immersed in 75% alcohol for 10 min for sterilization. The entire leg was carefully excised, minimizing blood loss to prevent contamination. The skin and muscle tissue were removed to fully expose the femur and tibia. Both ends of the femur or tibia were cut to allow insertion of a 1 mL syringe needle, and the red bone marrow was flushed out using complete medium (α-MEM supplemented with 10% fetal bovine serum [FBS], 1% streptomycin, and 50 ng/mL macrophage colony-stimulating factor [M-CSF]). The cell mass was gently triturated to obtain a single-cell suspension, which was then brought to the appropriate volume with the medium for culturing. The cell type and culture duration were appropriately labeled. After 3 days of culture, the medium was partially replaced, and on the fourth day, the cells were washed twice with phosphate-buffered saline (PBS). The adherent cells were collected as bone marrow-derived macrophages/monocytes (BMMs) for subsequent experiments.

### Osteoblasts extraction and culture

Neonatal C57BL/6J mice (1 day old) were immersed in 75% alcohol for 10 min. The entire calvaria was carefully excised, minced, and digested overnight in a solution of collagenase II (1 mg/mL in L-DMEM) in an incubator. The cell suspension was then gently triturated, centrifuged at 1500 rpm for 5 min, and the supernatant was discarded. The cell pellet was resuspended in the appropriate volume of medium (L-DMEM supplemented with 10% FBS and 1% streptomycin) for culture. Adherent cells from the first and second passages were collected as osteoblasts for further experiments.

### Osteoclast differentiation and staining

Bone marrow-derived macrophages (BMMs) were cultured in complete α-MEM. Recombinant RANKL protein (50 ng/mL, R&D Systems) was added to induce osteoclastogenesis. After 5 days of culture, cells were fixed with 4% paraformaldehyde (PFA) and stained with tartrate-resistant acid phosphatase (TRAP) solution at 37°C for 30 min. The samples were then processed for imaging analysis.

### Osteoblast differentiation and staining

Neonatal mouse calvarial osteoblasts were cultured in complete L-DMEM. Osteoblast differentiation medium (ODM), consisting of 10 nM dexamethasone, 50 μg/mL ascorbic acid, and 10 mM β-glycerophosphate, was added to promote osteoblastogenesis. Drugs were dissolved in DMSO. After 7 days, cells were fixed with 4% PFA and incubated overnight in ALP working solution (Beyotime Biotechnology). Cells were washed with PBS prior to imaging analysis. After 21 days, cells were again fixed with 4% PFA and incubated with Alizarin Red S solution (Ori Cell) for 20 min. The cells were washed with PBS before being processed for imaging analysis.

### Cell viability assay

BMMs and neonatal mouse calvarial osteoblasts were cultured in a 5% CO_2_, 37°C incubator. Once the cells had fully adhered, they were divided into drug-treated, control, and blank groups. After 24 and 48 h, a 10% CCK-8 solution was added to the α-MEM or L-DMEM medium in the dark, and cells were incubated at 37°C for 1 h. Optical density (OD) was measured at 450 nm using a microplate reader. Cell viability was calculated using the following formula:

$$\frac{{OD}_{drug\;group}-{OD}_{blank\;group}}{{OD}_{control\;group}-{OD}_{blank\;group}}\times 100\%.$$

### Western blot

BMMs were seeded in 6-well plates at a density of 5 × 10^5^ cells per well and allowed to adhere overnight. The cells were then starved in serum-free α-MEM for 2 h. Following starvation, cells were incubated with α-MEM, with or without EA/EB, for an additional 2 h, followed by incubation with RANKL for various time points (0, 5, 10, 20, 30, 60 min). Cells were washed twice with pre-chilled PBS and lysed with a cold lysis buffer (including protease inhibitor [50x], phosphatase inhibitor [50x], and PMSF [100x]) at 100 μL per well. Cells were scraped off using a cell scraper and transferred to pre-chilled 1.5 mL centrifuge tubes. Lysis was performed on ice for 15 min. The lysate was centrifuged at 12,000 rpm for 15 min, and the supernatant was transferred to a new pre-chilled 1.5 mL centrifuge tube. Protein concentration was determined using the BCA method following the manufacturer’s instructions. Samples were mixed with SDS-PAGE protein loading buffer (5x, Beyotime, P0286) and processed as instructed. Denatured proteins were separated by SDS-PAGE gel electrophoresis using the Trans-Blot^®^ Turbo™ Transfer System (Bio-Rad Laboratories, Hercules, CA, United States) and transferred to a nitrocellulose membrane. The blot was blocked with TBST containing 5% (w/v) skim milk powder for 1 h with gentle agitation. The membrane was then incubated with primary antibodies (dilution ratio as specified) overnight at 4°C. After washing with TBST, secondary antibodies (dilution ratio as specified) were added and incubated for 1 h at room temperature. The blot was washed three times with wash buffer, each for 5 min. Imaging was performed using a ChemiDoc™ MP imaging system (Bio-Rad Laboratories, Hercules, CA, United States).

### Quantitative real-time PCR (qRT-PCR)

Osteoclasts were seeded in 12-well plates at a density of 2 × 10^5^ cells per well and cultured overnight in induction medium, with or without EA/EB. RNA was extracted from the cells using Trizol reagent, following the manufacturer’s protocol. Reverse transcription was performed using the Prime Script™ RT Master Mix (Perfect Real Time, Takara RR036B) according to the provided instructions, resulting in the synthesis of cDNA. Quantitative real-time PCR was conducted using TB Green Premix Ex Taq on a qTOWER^3^ Real-time PCR thermal cycler, PCR reaction conditions are shown in Table 1 and the primer sequences for qRT-PCR are shown in Table 2.

**TABLE 1:** PCR reaction conditions.

Step	Program	Time	Cycles
Initial Denaturation	95°C	5 min	1
Denaturation	95°C	10 s	30
Annealing	50°C–72°C	20 s	
Extension	72°C	20 s	
Final Extension	72°C	10 min	1
Hold	4°C	∞	1

GAPDH, was used as the internal reference, and the relative expression levels of the samples were calculated using the 2^−ΔΔCT^, method.

**TABLE 2:** The primer sequences for qRT-PCR.

Gene name	Forward primer (5′-3′)	Reverse primer (3′-5′)
c-Fos	GCGAGCAACTGAGAAGAC	TTGAAACCCGAGAACATC
NFATc1	CAA​CGC​CCT​GAC​CAC​CGA​TAG	GGC​TGC​CTT​CCG​TCT​CAT​AGT
Acp5	TGTGGCCATCTTTATGCT	GTCATTTCTTTGGGGCTT
CTSK	CTT​CCA​ATA​CGT​GCA​GCA​GA	TCT​TCA​GGG​CTT​TCT​CGT​TC
TRAP	CTG​GAG​TGC​ACG​ATG​CCA​GCG​ACA	TCC​GTG​CTC​GGC​GAT​GGA​CCA​GA
ATP6V0d2	AAG​CCT​TTG​TTT​GAC​GCT​GT	TTC​GAT​GCC​TCT​GTG​AGA​TG
Ctr	GAG​GTT​CCT​TCT​CGT​GAA​CAG	AGT​CAG​TGA​GAT​TGG​TAG​GAG​C
Ctsk	CTT​CCA​ATA​CGT​GCA​GCA​GA	TCT​TCA​GGG​CTT​TCT​CGT​TC
MMP9	AGC​CGA​CTT​TTG​TGG​TCT​TC	AGA​CTG​CTT​CTC​TCC​CAT​CA
Dcstamp	TCC​TCC​ATG​AAC​AAA​CAG​TTC​CAA	AGA​CGT​GGT​TTA​GGA​ATG​CAG​CTC
Itgb3	GAA​GGA​GTG​TGT​GGA​GTG​TAA​GA	GTT​TTT​GCC​AGT​ATC​CGT​CAG​CTC
Collagentype1	CGA​CCT​CAA​GAT​GTG​CCA​CT	GCA​GTA​GAC​CTT​GAT​GGC​GT
ALPl	CCA​ACT​CTT​TTG​TGC​CAG​AGA	GGC​TAC​ATT​GGT​GTT​GAG​CTT​TT
Osteocalcin	CAG​AAC​AGA​CAA​GTC​CCA​CAC​A	TCA​GCA​GAG​TGA​GCA​GAA​AGA​T
Runx2	GAC​TGT​GGT​TAC​CGT​CAT​GGC	ACT​TGG​TTT​TTC​ATA​ACA​GCG​GA
Ncadherin	CCA​TCC​TGA​CAG​ACC​CCA​AC	ACT​GAG​GTG​GGT​GCT​GAA​TG
FAK	CCA​TGC​CCT​CGA​AAA​GCT​ATG	TGA​CGC​ATT​GTT​AAG​GCT​TCT
PYK2	CTT​GCC​GTG​TTC​CCT​GTA​GT	CGC​CAC​TCC​CAA​ACC​TAC​TT
GAPDH	AGG​TCG​GTG​TGA​ACG​GAT​TTG	GGG​GTC​GTT​GAT​GGC​AAC​A

### NF-κb luciferase assay

RAW 264.7 cells were seeded in 48-well plates at a density of 60% (5-10 × 10^5^ cells) with 500 μL of medium (α-MEM supplemented with 10% FBS and 1% streptomycin) per well. Once the cell density reached 80% after overnight incubation, cells were treated with drug solutions prepared in complete medium for 1 h, followed by stimulation with RANKL and NF-κB for 6 h. Specifically, 250 μL of the drug solution was added to each well initially, and an additional 250 μL containing both the drug and RANKL was added after 1 h. After treatment, 100 μL of lysis buffer was added to each well, and the lysates were collected into 1.5 mL Eppendorf tubes. The samples were centrifuged at 14,000 rpm for 20 min at 4°C. The supernatant was transferred to new 1.5 mL Eppendorf tubes, with 50 μL of each supernatant added to a white 96-well plate for analysis; the remaining supernatant was stored at −20°C. To each well, 50 μL of luciferase detection solution was added, avoiding bubbles. Chemiluminescence was detected using a microplate reader, and the measured values represented NF-κB activity.

### Network pharmacology

#### Construction of a chemical database of epimedium

To systematically compile information on the names, structures, CAS numbers, and classifications of compounds within *Epimedium*, a search was conducted across several databases, including TCMID (http://www.megabionet.org/tcmid), TCM Database@Taiwan (http://tcm.cmu.edu.tw), and the Chemistry Database (http://www.chemcpd.csdb.cn/scdb). This facilitated the establishment of an internal chemical database. A comprehensive literature review, supplemented by information from books, the Encyclopedia of Chinese Medicine, and the PubChem database (https://pubchem.ncbi.nlm.nih.gov), was conducted to validate, integrate, and augment the chemical data.

#### Construction of a database with known anti-osteoporosis compounds in epimedium

A targeted search was performed in the PubMed database (https://pubmed.ncbi.nlm.nih.gov) using keywords combining the active compound names in *Epimedium* with terms related to osteoporosis, bone loss, bone mineral density, osteoclastogenesis, or osteoclasts. Compounds exhibiting anti-osteoporosis activity were identified and summarized.

#### Construction of a potential active compound interaction network based on molecular similarity calculation

The SMILES format of compounds in the chemical database was converted into SDF format using the Open Babel tool (O'Boyle et al., 2011). The Rdkit package (https://www.rdkit.org/) in Python (https://www.python.org/) was then used to compute the Tanimoto similarity between compounds with known anti-osteoporosis activity and those in *Epimedium*, using ECFP4 circular topological fingerprints. The results were stored in an Excel file using the pandas package. Based on previous analyses suggesting a Tanimoto similarity threshold of 0.4 for ECFP4 fingerprints, our study set the threshold at 0.5 to enhance precision (Muegge and Mukherjee, 2016). A compound–compound interaction network was constructed using Cytoscape 3.7.2, connecting compounds with a Tanimoto similarity greater than 0.5 to outline potential associations.

#### Preliminary screening of potential target proteins

From the screening results, a clustering analysis based on molecular structures of potential active ingredients was conducted. The top five compounds from each cluster were selected for preliminary cytological validation. Standard samples of all active ingredients were obtained, and *in vitro* experiments were conducted using an osteoclast system to identify compounds that significantly inhibited osteoclast differentiation and maturation.

#### Target prediction of drugs

The potential targets of the drugs were predicted using Pharm Mapper (Pharm Mapper (lilab-ecust.cn)), the Similarity Ensemble Approach (SEA Search Server (bkslab.org)), Swiss Target Prediction (Swiss Target Prediction), and Super Pred (https://prediction.charite.de). The union and intersection of predicted targets from these websites were analyzed using the Ingenuity Pathway Analysis (IPA) database to determine the drug targets.

#### Molecular docking

Molecular docking was performed using Autodock 4.2.6 software. The crystal structures of FAK1 (AF-P34152-F1) and FAK2 (AF-Q9QVP9-F1) were obtained from the UniProt database. The 3D structures of small molecules such as Epimedin A and Epimedin B were downloaded from the PubChem Compound database. Protein structures were prepared by removing water, adding hydrogens, computing Gasteiger charges, and assigning AD4 types. Small molecules were initialized by adding Gasteiger charges, incorporating non-polar hydrogens, and setting up rotatable bonds, then converted to PDBQT format. The docking grid box was set to encompass the FAK1 and FAK2 proteins. Docking was conducted with 100 runs using a genetic algorithm (GA) with default parameters. The lowest energy conformation was selected as the final docking result. PyMOL software was used to visualize the docking outcomes. Effective docking was defined as having a root mean square deviation of binding energy less than 2, with a binding energy less than 0 kcal/mol indicating potential natural state binding, and less than −1.2 kcal/mol considered a strong binding affinity. The final 3D interaction structures were generated using the PLIP website (PLIP - Welcome (tu-dresden.de)) and PyMOL, while 2D interaction structures were created using the Proteins Plus Server (Zentrum für Bioinformatik: Universität Hamburg - Proteins Plus Server).

#### Statistical analysis

Statistical analyses were conducted using ImageJ software and GraphPad Prism 7.0. Independent sample t-tests were used to compare differences between two groups. P-values or adjusted P-values less than 0.05 were considered statistically significant.

## Results

### Construction of a database of known anti-osteoporosis active ingredients in *epimedium*

We constructed a comprehensive database comprising 108 unique compounds isolated from *Epimedium*, as detailed in Supplementary Table S1. Among these, 45 compounds demonstrated active effects on bone and cartilage formation in both *in vivo* and *in vitro* studies. Notably, eight of these compounds were the focus of target-specific studies, while the remaining compounds were examined primarily in pathway-related research.

### Construction of an active component network of *epimedium* based on molecular similarity

**FIGURE 1:**
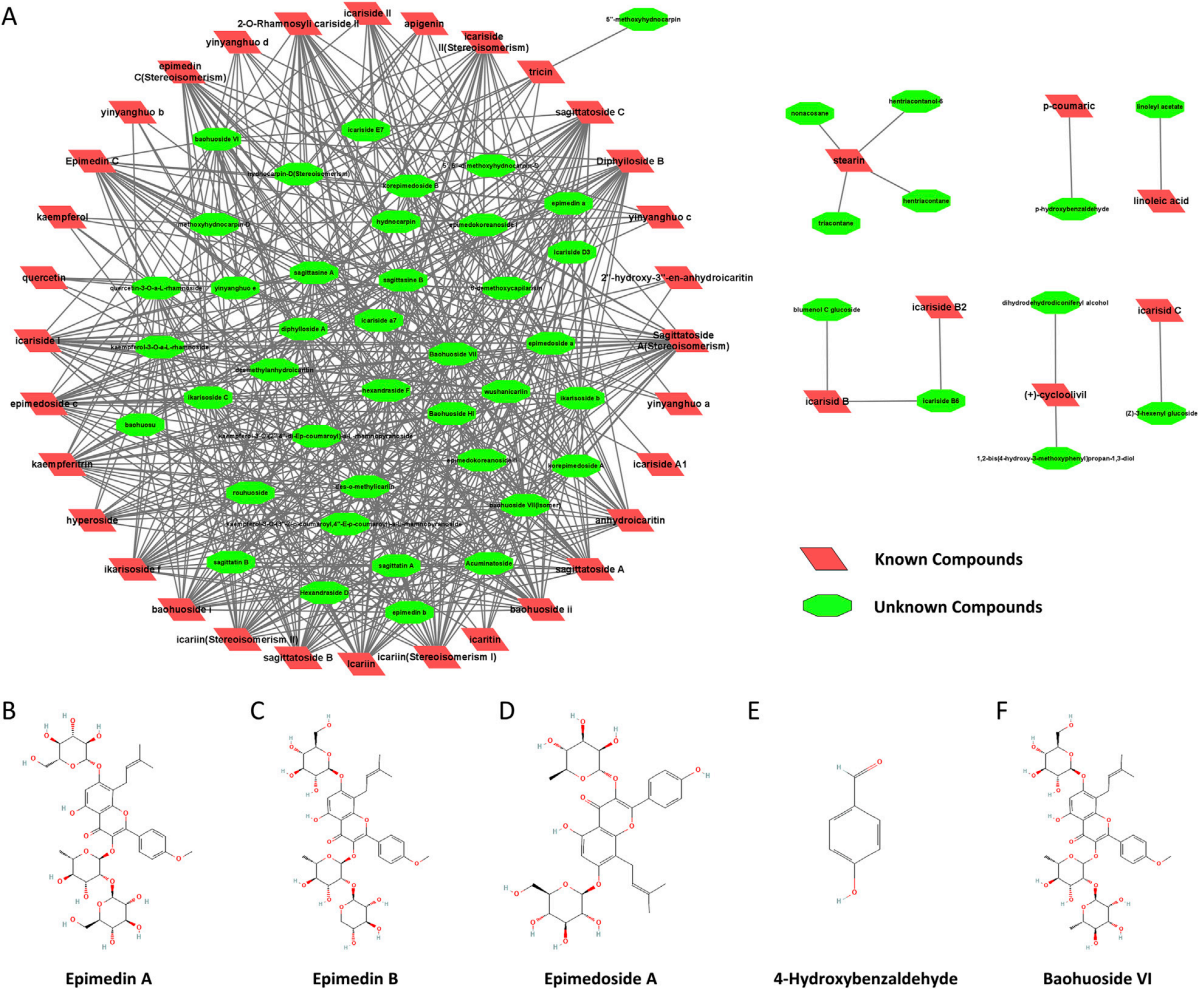
Construction of the active component network of *Epimedium* based on molecular similarity. **(A)**. Similarity between candidate compounds and known active compounds. B-F. Chemical structure diagrams (National Center for Biotechnology Information (2024). Retrieved 6 February 2024, from PubChem): **(B)** Epimedin A (PubChem Compound Summary for CID 13916051). **(C)** Epimedin B (PubChem Compound Summary for CID 5748393). **(D)** Epimedoside A (PubChem Compound Summary for CID 5317093). **(E)** 4-Hydroxybenzaldehyde (PubChem Compound Summary for CID 126). **(F)** Baohuoside VI (PubChem Compound Summary for CID 5488005).

To further understand the active component network of *Epimedium*, we performed a structural similarity analysis, identifying seven major substance classes. The degree of similarity between candidate compounds and known active compounds is depicted in Figure 1. Using this data, we calculated and ranked the average similarity scores of candidate compounds within the network, applying a screening criterion of a similarity threshold greater than 0.4. Table 1 presents these results, with the seventh class of compounds showing the highest abundance. From this class, the top ten compounds were selected, ensuring structural diversity and availability for experimental validation.

### Optimal drug concentration (drug cytotoxicity)

**FIGURE 2:**
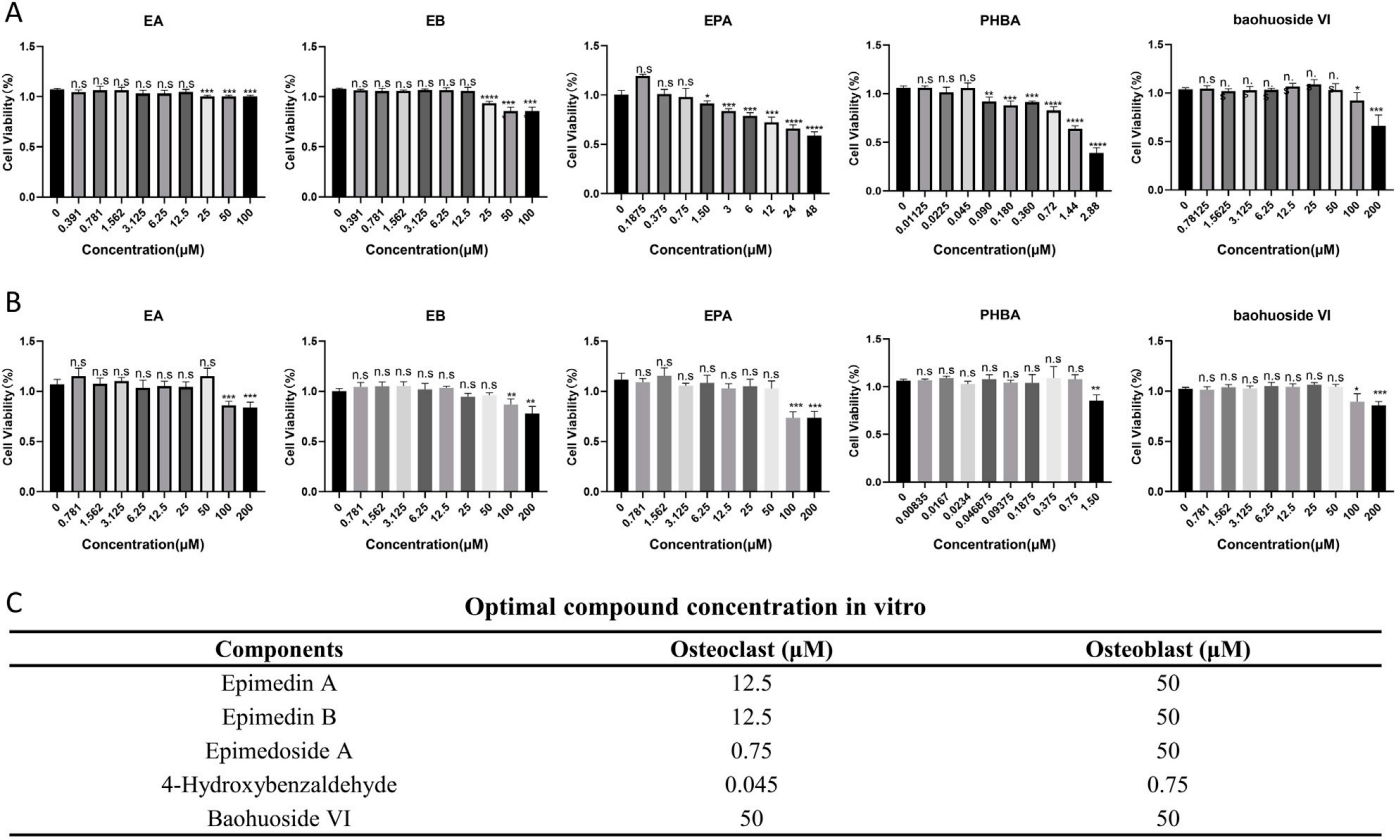
Optimal compound concentration screening *in vitro*. **(A)** Effects of compounds on the viability of bone marrow-derived macrophages (BMMs) after 72 h (n = 4). **(B)** Effects of compounds on the viability of precalvarial osteoblasts after 72 h (n = 4). *P < 0.05; **P < 0.01; ***P < 0.001; ****P < 0.0001. **(C)** Optimal compound concentration *in vitro*.

The optimal concentrations of five selected compounds were determined using primary osteoclasts and osteoblasts. Through the CCK8 assay, it was observed that 12.5 μM for EA and EB, 0.75 μM for EPA, 0.045 μM for PHBA, and 50 μM for baohuoside VI exhibited the most favorable effects on primary osteoclast BMMs. In contrast, for primary osteoblast calvarial precursors, the most effective concentrations were 50 μM for EA, EB, and EPA, 0.75 μM for PHBA, and 50 μM for baohuoside VI (Figures 2A–C). These findings indicate that EPA and PHBA exhibit higher toxicity compared to the other three compounds, particularly in BMMs.

### EA and EB inhibit RANKL-Induced osteoclast differentiation in BMMs

**FIGURE 3:**
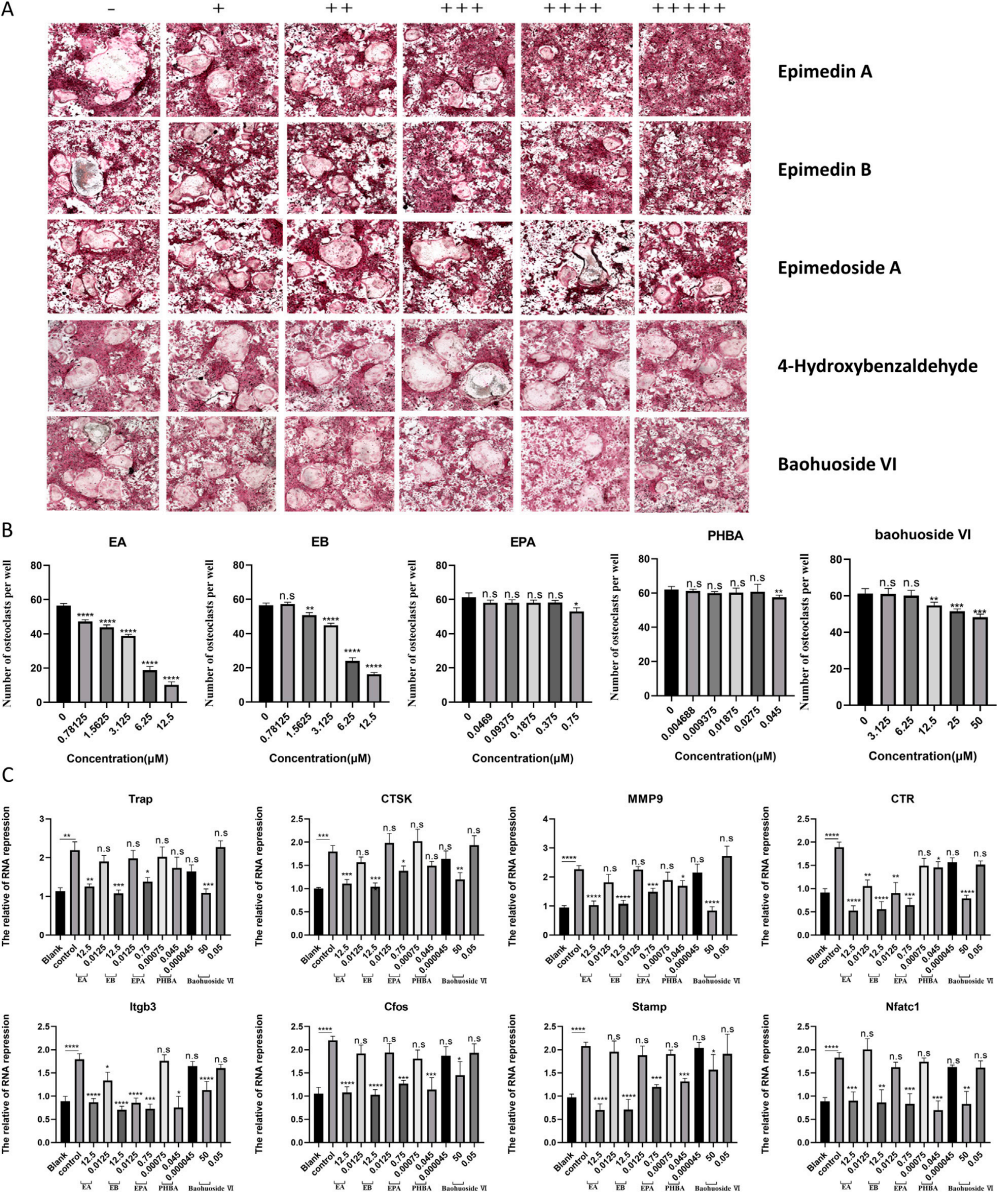
Compounds suppress RANKL-induced osteoclast formation without cytotoxicity *in vitro*. **(A)** BMMs were stimulated with RANKL in the presence of varying concentrations of EA (0, 0.78125, 1.5625, 3.125, 6.25, 12.5 μM), EB (0, 0.78125, 1.5625, 3.125, 6.25, 12.5 μM), EPA (0, 0.0469, 0.09375, 0.1875, 0.375, 0.75 μM), PHBA (0, 4.6875, 9.375, 18.75, 27.5, 45 nM), and Baohuoside VI (0, 3.125, 6.25, 12.5, 25, 50 μM), followed by TRAP staining. **(B)** Quantitative analysis of TRAP-positive multinucleated cells (nuclei ≥3). **(C)** BMMs were cultured with M-CSF (30 ng/mL) and RANKL (50 ng/mL) in the presence of the indicated concentrations of the compounds. Expression of osteoclast-specific genes (Trap, Ctsk, Mmp9, Ctr, Itgb3, C-fos, Stamp, Nfatc1) was assessed by qRT-PCR. Gene expression levels were normalized to GAPDH. *P < 0.05; **P < 0.01; ***P < 0.001; ****P < 0.0001 compared to the control group.

In our experiments, BMMs were incubated with M-CSF (30 ng/mL) and RANKL (100 ng/mL) to induce osteoclastogenesis. Notably, EA and EB significantly inhibited RANKL-induced osteoclastogenesis in a dose-dependent manner, without showing cytotoxicity. In contrast, EPA, PHBA, and baohuoside VI did not exhibit a dose-dependent inhibition of RANKL-induced osteoclastogenesis (Figures 3A,B). To further elucidate the effects of these compounds on osteoclast differentiation, we analyzed the expression of osteoclast-specific genes using quantitative PCR. The results showed that the expression of osteoclast marker genes, including Trap, Ctsk, Mmp9, Ctr, Itgb3, C-fos, Stamp, and Nfatc1, was upregulated in the RANKL-induced control group. However, treatment with EA and EB led to a repression of these gene expressions during osteoclastogenesis (Figure 3C). These findings suggest that EA and EB suppress osteoclastogenesis by specifically inhibiting RANKL-induced expression of osteoclast-specific genes, providing insights into the molecular mechanisms underlying the inhibitory effects of EA and EB on osteoclast differentiation.

### EA and EB induce osteoblast differentiation in the presence of osteoactivin (OA)

**FIGURE 4:**
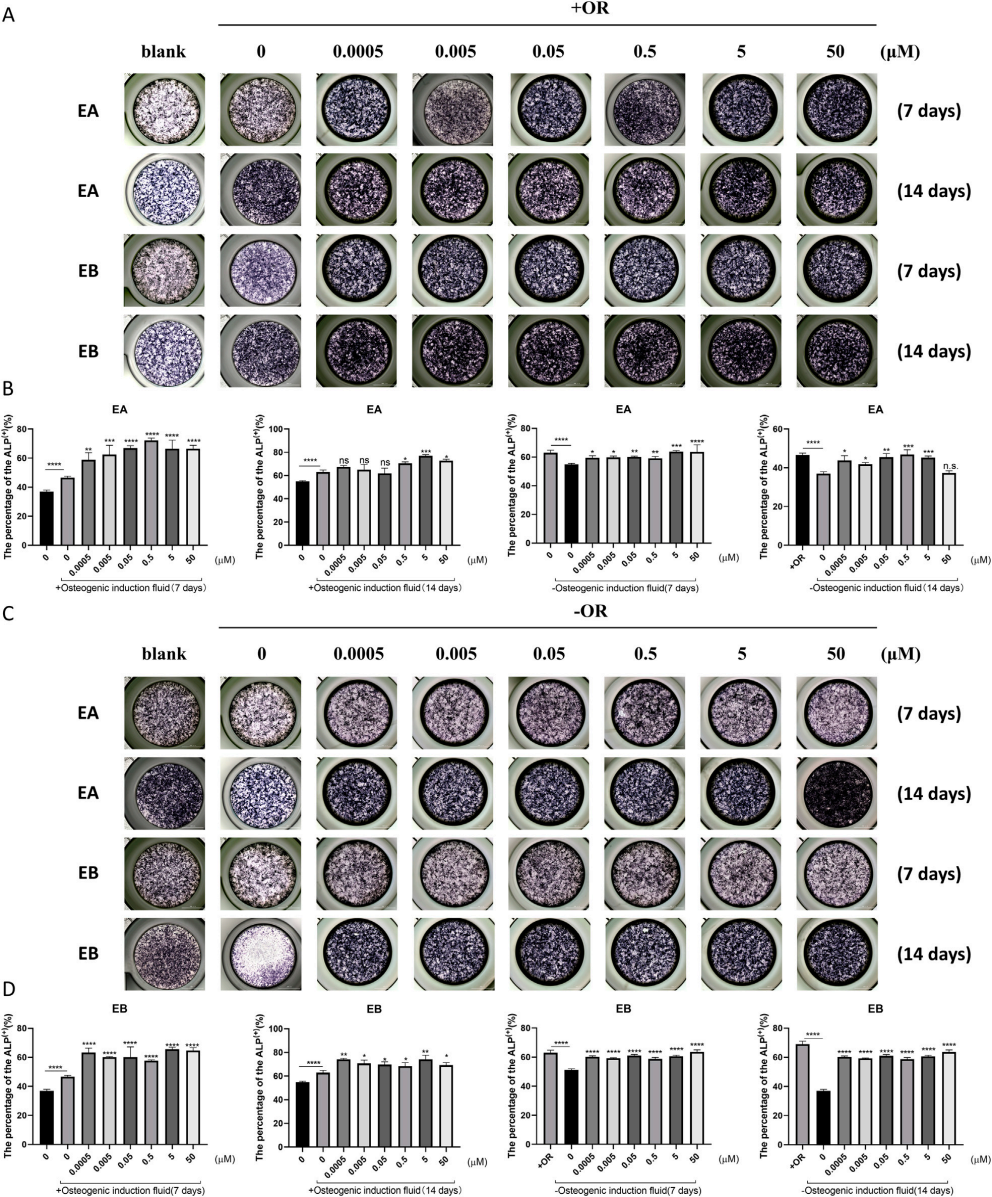
EA and EB promote osteoblast formation without cytotoxicity *in vitro*. **(A)** Cranial osteoblasts were stimulated with osteogenic induction medium (OR) in the presence of various concentrations of EA (0, 0.0005, 0.005, 0.05, 0.5, 5, 50 μM) and EB (0, 0.0005, 0.005, 0.05, 0.5, 5, 50 μM), followed by ALP staining at 7 and 14 days. **(C)** Cranial osteoblasts were stimulated without osteogenic induction medium (OR) under the same conditions. **(B,D)** Quantitative analysis of ALP-positive cells. *P < 0.05; **P < 0.01; ***P < 0.001; ****P < 0.0001 compared to the control group.

**FIGURE 5:**
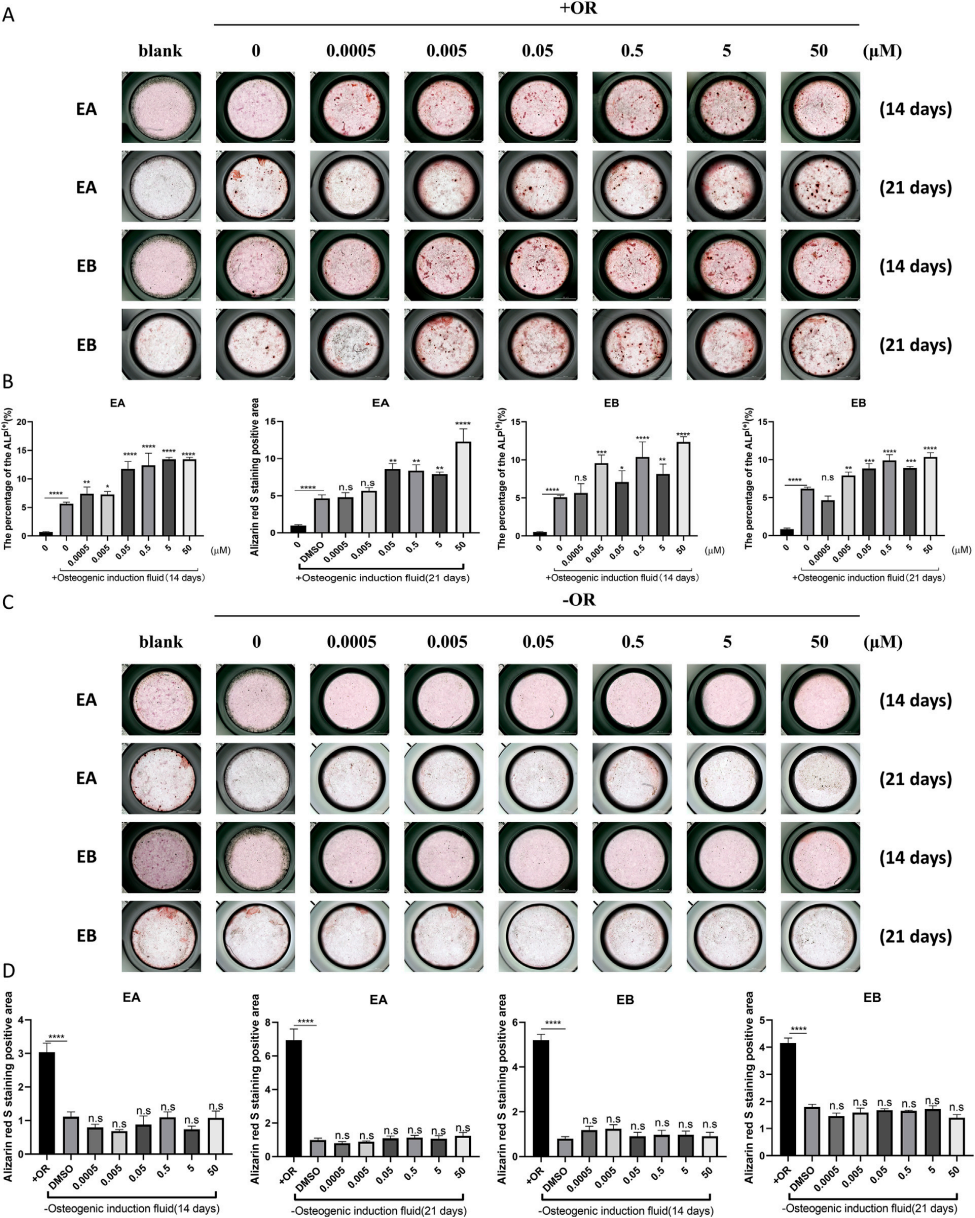
EA and EB enhance calcium deposition in osteoblasts *in vitro*. Cranial osteoblasts were stimulated with **(A)** and without **(C)** osteogenic induction medium (OR) in the presence of varying concentrations of EA (0, 0.0005, 0.005, 0.05, 0.5, 5, 50 μM) and EB (0, 0.0005, 0.005, 0.05, 0.5, 5, 50 μM). Cells were stained with Alizarin Red for calcium detection at 14 and 21 days. **(B,D)** Quantitative analysis of Alizarin Red-positive cells. *P < 0.05; **P < 0.01; ***P < 0.001; ****P < 0.0001 compared to the control group.

**FIGURE 6:**
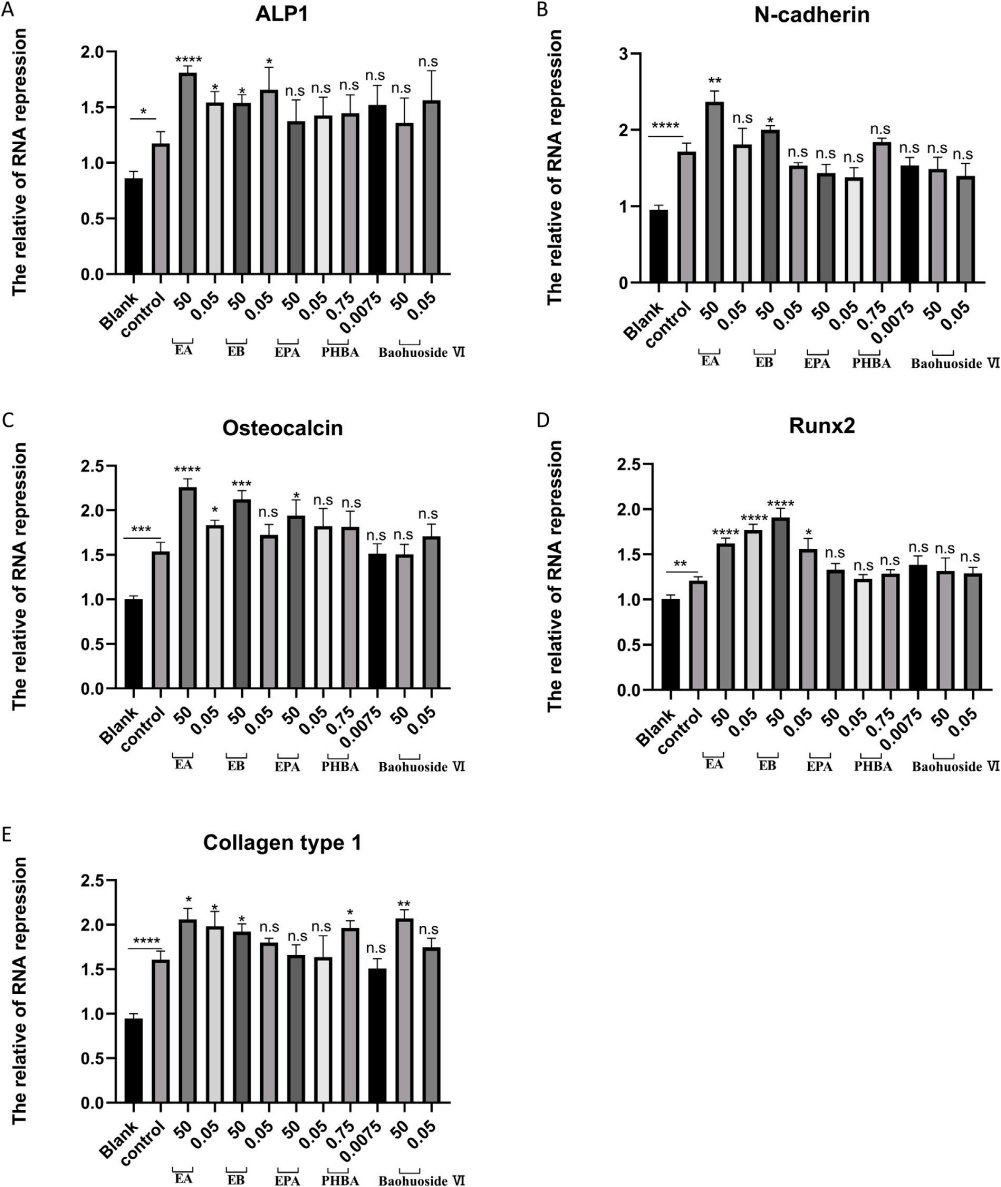
EA and EB regulate the expression of osteoblast-related genes. Cranial osteoblasts were stimulated with osteogenic induction medium (OR) in the presence of different concentrations of EA (50 μM and 0.05 μM), EB (50 μM and 0.05 μM), EPA (50 μM and 0.05 μM), Baohuoside VI (50 μM and 0.05 μM), and PHBA (0.75 μM and 0.00075 μM) for 7 days. Gene expression levels of ALP1 **(A)** N-cadherin **(B)** osteocalcin **(C)** RUNX2 **(D)** and collagen type I **(E)** were measured. Expression levels were normalized to GAPDH. *P < 0.05; **P < 0.01; ***P < 0.001; ****P < 0.0001 compared to the control group.

Bone homeostasis is maintained by the coordinated activity of osteoclasts and osteoblasts. After establishing the inhibitory effects of EA and EB on osteoclasts, we explored their potential role in promoting osteogenesis. We examined whether these compounds could induce the differentiation of primary mouse precalvarial osteoblasts in the presence or absence of osteogenic induction medium. ALP staining results revealed that EA and EB enhanced ALP production in a dose- and time-dependent manner when combined with osteogenic induction medium. Higher concentrations and longer exposure times resulted in more intense ALP staining (Figures 4A,B). In contrast, the other three drugs only induced alkaline phosphatase activity at high concentrations (Supplementary Figure S1A, B, Supplementary Figure S2A, B). Without osteogenic induction medium, only EA, EB, and EPA maintained the ability to induce ALP activity (Figures 4C,D; Supplementary Figures S2A, B), while the other two compounds had minimal effects (Supplementary Figures S1C, D). Calcium salt deposition, evaluated through Alizarin Red staining, showed that EA, EB, and EPA promoted calcium deposition, with more significant effects at higher concentrations (Figures 5A,B; Supplementary Figures S2C, D). The other two drugs showed comparatively weaker effects (Supplementary Figures S3A, B). Administration of drugs alone, without osteogenic induction medium, did not enhance calcium deposition (Figures 5C,D; Supplementary Figures S2C, D, Supplementary Figures S3C, D). Quantitative PCR analysis of osteogenesis-related gene expression revealed that EA and EB significantly upregulated the expression of RUNX2, osteocalcin, ALP1, N-cadherin, and collagen type I, while the other three compounds did not (Figures 6A–E). These results indicate that EA and EB are potent promoters of osteoblast differentiation and function, while the other compounds have limited effects.

### EA and EB alleviate OVX-Induced bone loss in vivo

**FIGURE 7:**
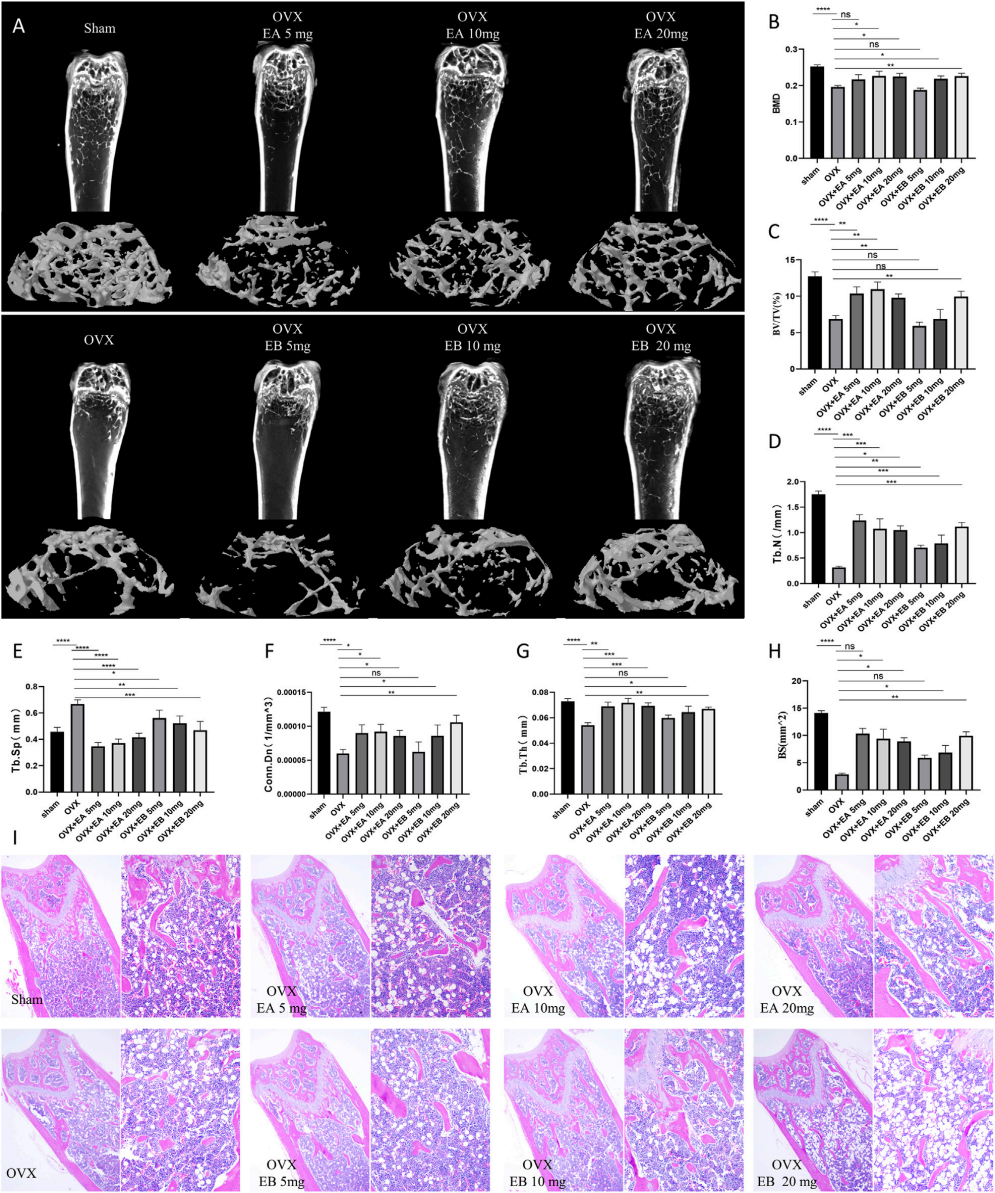
EA and EB attenuate OVX-induced bone loss *in vivo*. **(A)** Representative micro-CT images of the distal femur from sham-treated controls (Sham), OVX with PBS injection (OVX), and OVX with EA or EB injections at doses of 5 mg/kg, 10 mg/kg, and 20 mg/kg. **(B)** Quantitative assessment of bone mineral density (BMD). **(C)** Bone volume/tissue volume (BV/TV). **(D)** Trabecular number (Tb.N). **(E)** Trabecular Separation/Spacing (Tb.Sp). **(F)** Connection density (Conn.Dn). **(G)** Trabecular thickness (Tb.Th). and **(H)** Bone surface (BS) in each group (n = 5). **(I)** Representative images of H&E-stained sections from each treatment group. Data are presented as mean ± SD. *P < 0.05; **P < 0.01; ***P < 0.001; ****P < 0.0001 compared to the OVX group.

Given the observed inhibitory effects of EA and EB on osteoclastogenesis and their promotion of osteoblastogenesis *in vitro*, we investigated their therapeutic potential in an OVX-induced bone loss murine model. Micro-CT analysis revealed that EA and EB mitigated bone mass loss in the distal femur of OVX mice in a dose-dependent manner compared to the OVX control group (Figure 7A). Quantitative analysis showed significant improvements in BMD, Tb.N, BV/TV, Conn.Dn, Bs, and Tb.Th, and reductions in Tb.Sp, even at low doses of EA or EB (5 mg/kg) (Figures 7B-H). Histological evaluation confirmed that EA and EB administration every other day attenuated OVX-induced bone loss, with no observed cytotoxicity in multiple organ sections at high doses (20 mg/kg) (Figures 7C-I; Supplementary Figure S4A). TRAP staining corroborated the micro-CT findings, showing a significant decrease in TRAP-positive cells around trabeculae in EA or EB-treated groups compared to OVX mice (Supplementary Figures S4B, C). These results collectively demonstrate that EA and EB effectively inhibit osteoclast formation and prevent OVX-induced bone loss.

### EA and EB do not inhibit RANKL-Induced activation of MAPK and NF-κB signaling pathways

**FIGURE 8:**
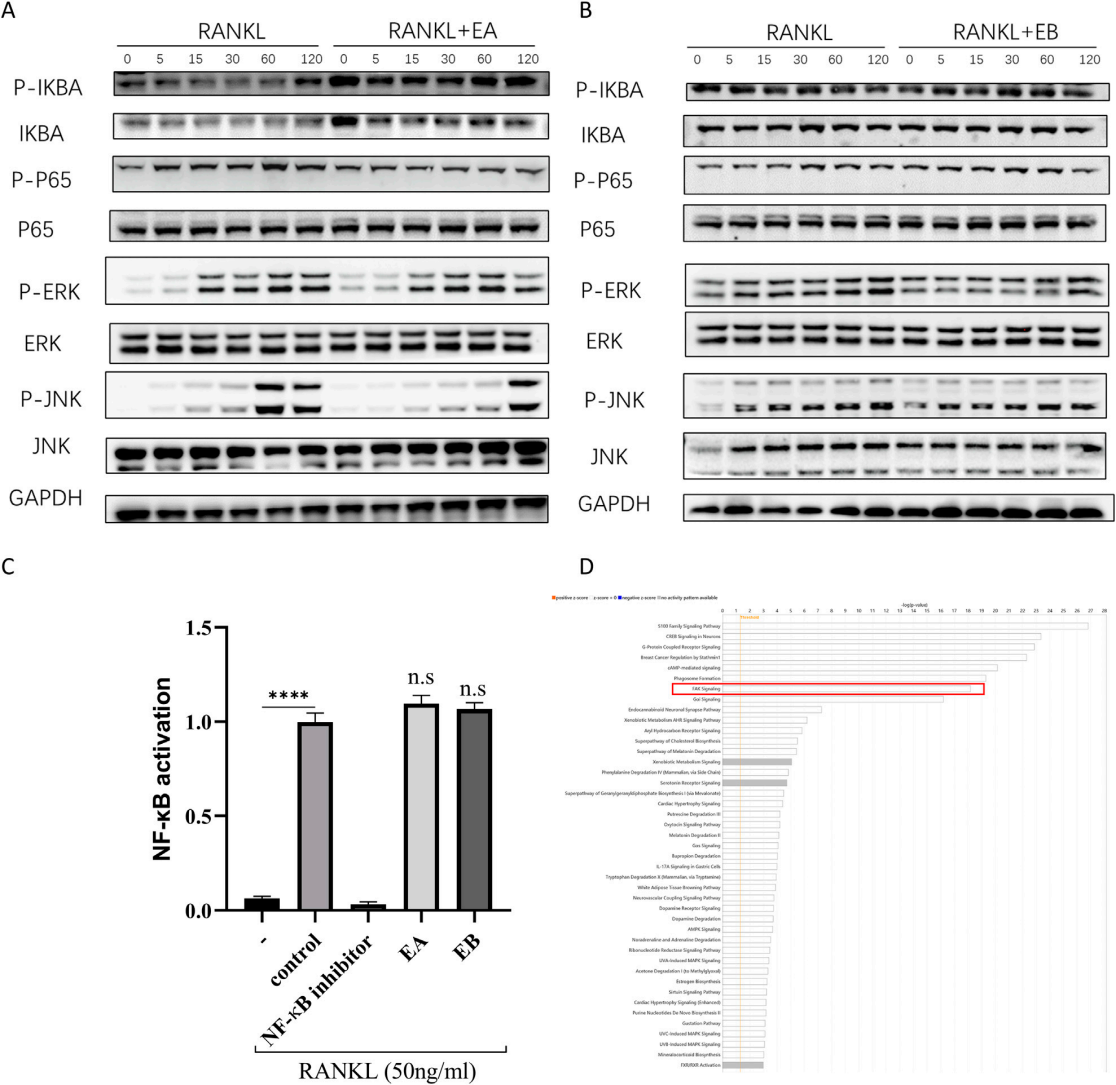
EA and EB do not regulate RANKL-induced activation of MAPK and NF-κB signaling pathways. **(A,B)** BMMs were pretreated with or without EA or EB in serum-free medium for 2 h, followed by stimulation with RANKL (100 ng/mL). The activities of the NF-κB and MAPK signaling pathways were assessed by Western blot analysis using specific antibodies against p-ERK, ERK, p-JNK, JNK, P-P65, P65, p-IκBα, and IκBα. GAPDH served as the loading control. **(C)** RAW 264.7 cells were used for NF-κB luciferase reporter assay after 7 h of stimulation with EA and EB (12.5 μM). ****P < 0.0001. **(D)** Ingenuity Pathway Analysis (IPA) was utilized to analyze the combined target prediction results from PharmMapper, Similarity Ensemble Approach, SwissTargetPrediction, and SuperPred, identifying the targeted signaling pathways of the compounds.

To investigate the mechanisms by which EA and EB suppress osteoclast differentiation, we examined the activation of early MAPK and NF-κB signaling cascades in response to RANKL. Western blot analysis showed that RANKL-induced phosphorylation of ERK and JNK, indicative of MAPK activation, was not attenuated by EA or EB treatment (Figures 8A,B). Similarly, RANKL-induced IκBα degradation and p65 phosphorylation, markers of NF-κB activation, were unaffected by EA or EB (Figures 8A,B). The NF-κB luciferase reporter assay further confirmed that EA and EB did not significantly inhibit NF-κB activation (Figure 8C). These findings suggest that the suppression of osteoclast differentiation by EA and EB is not mediated through the MAPK or NF-κB signaling pathways, indicating that other signaling pathways may be involved.

### EA and EB May regulate osteoblast and osteoclast differentiation through the FAK signaling pathway

Since EA and EB did not affect MAPK and NF-κB pathways, we hypothesized that other signaling pathways might be involved in their regulation of osteoclast and osteoblast differentiation. Using target prediction tools such as PharmMapper, SEA Search Server, SwissTargetPrediction, and SuperPred, we identified potential targets of EA and EB (Supplementary File S1). Ingenuity Pathway Analysis (IPA) indicated that the FAK signaling pathway was a potential target of EA and EB (Figure 8D).

### Molecular docking analysis

**FIGURE 9:**
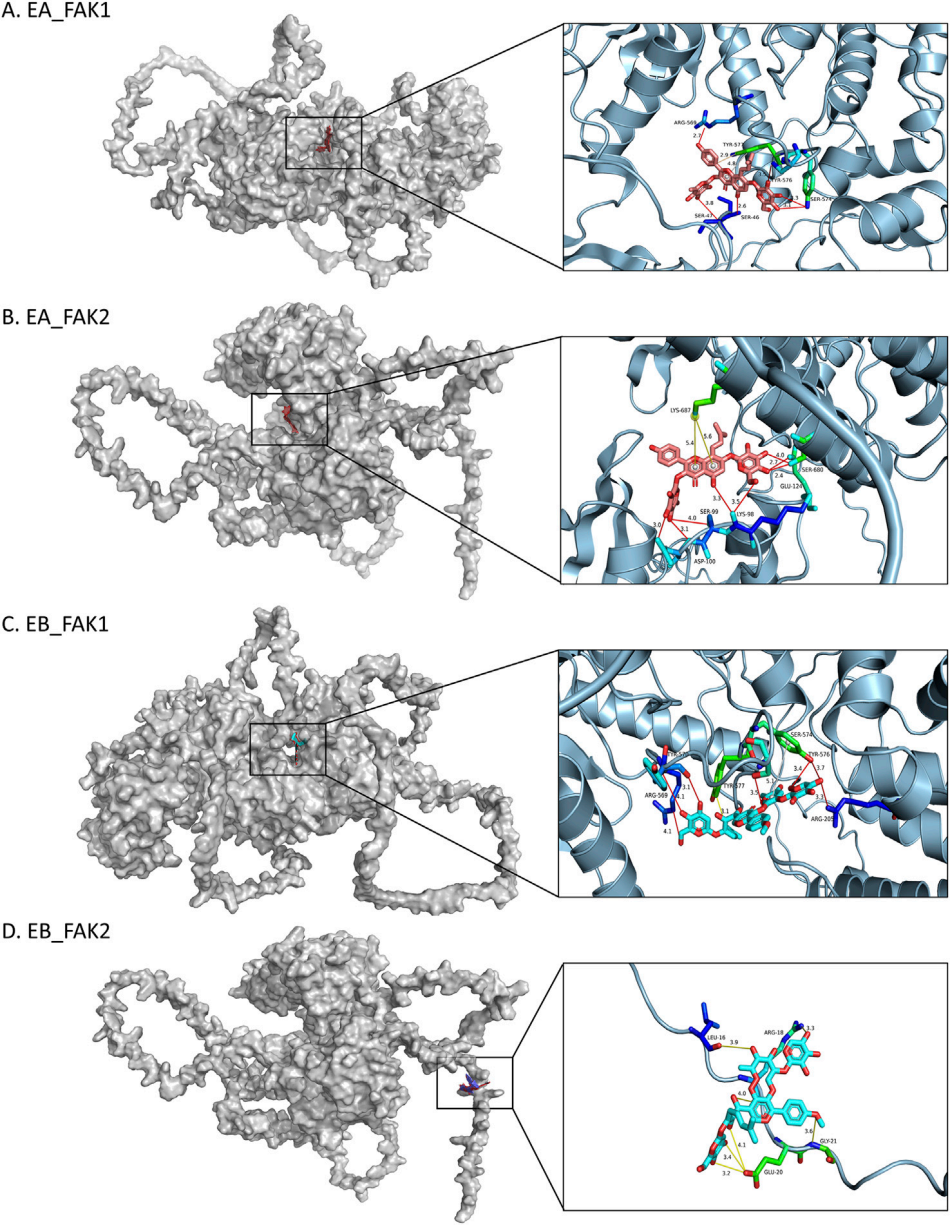
Molecular docking models of FAK1 and FAK2. 3D images showing the binding sites of: **(A)** FAK1 with EA, **(B)** FAK2 with EA, **(C)** FAK1 with EB, **(D)** FAK2 with EB. In the 3D images, interacting amino acids are depicted as sticks. EA is shown as pink sticks, and EB as blue sticks. Hydrogen bonds are indicated by yellow lines.

**FIGURE 10:**
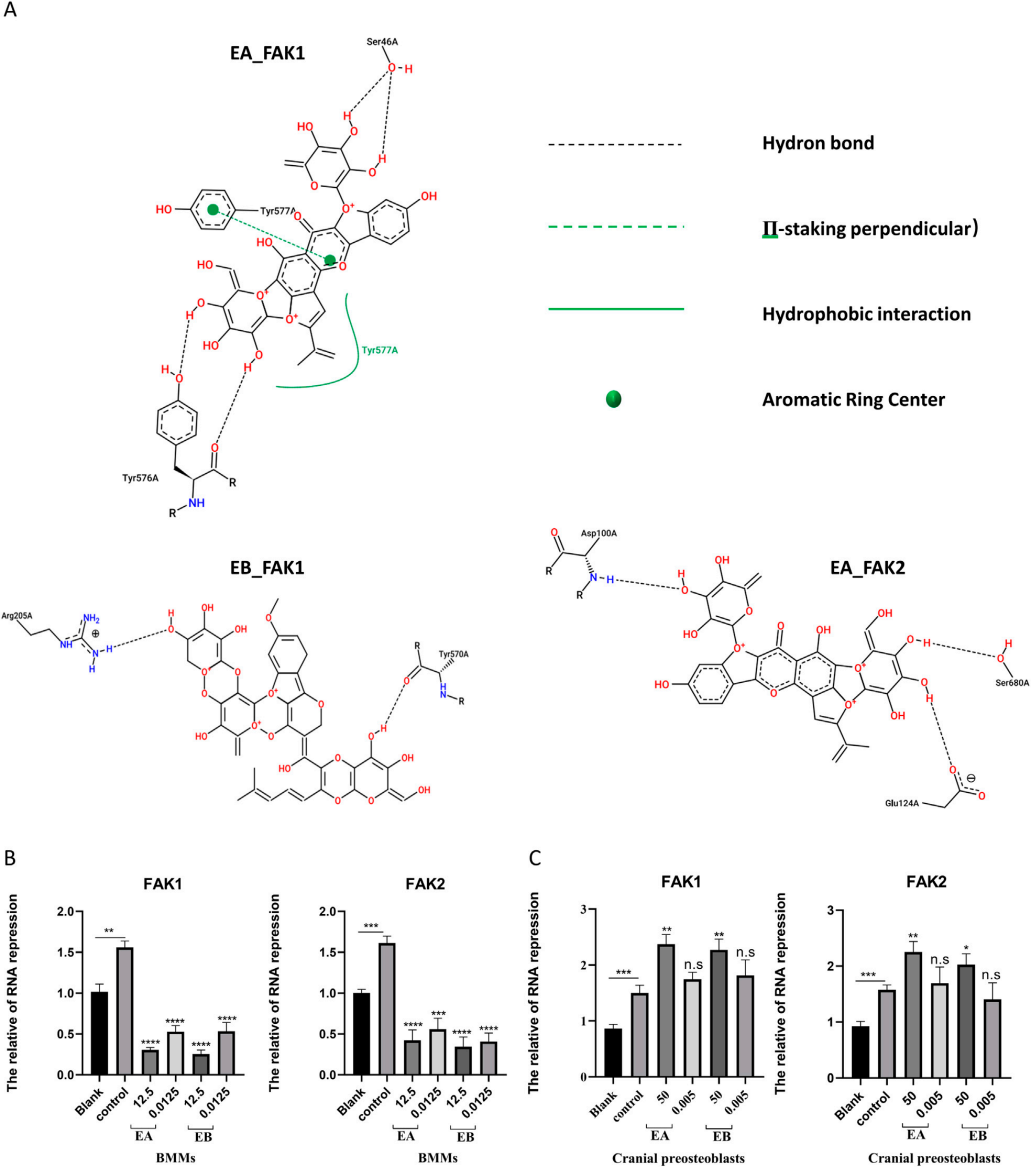
**(A)** The 2D interaction structures between the compounds and proteins were generated using the Zentrum für Bioinformatik website. **(B)** BMMs were stimulated with M-CSF (30 ng/mL) and RANKL (50 ng/mL) in the presence of EA (12.5 μM and 0.0125 μM) and EB (12.5 μM and 0.0125 μM) for 7 days. **(C)** Cranial osteoblasts were stimulated with osteogenic induction medium (OR) in the presence of different concentrations of EA (50 μM and 0.05 μM) and EB (50 μM and 0.05 μM) for 7 days. Expression levels of FAK1 and FAK2 were examined by qRT-PCR. Gene expression levels were normalized to GAPDH. **P < 0.01; ***P < 0.001; ****P < 0.0001.

To predict the binding interactions between EA, EB, and the target proteins FAK1 (AF-P34152-F1) and FAK2 (AF-Q9QVP9-F1), molecular docking was performed. Docking results showed that EA binds effectively to the hydrophobic pocket of FAK1, forming hydrogen bonds with residues SER47, ARG569, SER574, and TYR576; π-stacking with TYR577; and hydrophobic interactions with TYR577, with a minimum binding energy of −13.012 kJ/mol (Figure 9A). EA also demonstrated binding to FAK2, with hydrogen bonds involving residues SER99, ASP100, GLU124, and SER680, and a π-cation interaction with LYS687, yielding a minimum binding energy of −5.815 kJ/mol (Figure 9B). Similarly, EB bound effectively to FAK1, interacting with residues ARG205, ARG569, TYR570, SER574, and TYR576, with a minimum binding energy of −14.0164 kJ/mol (Figure 9C). EB also showed binding to FAK2, forming hydrogen bonds with ARG18, GLU20, and GLY21, with a binding energy of −6.4852 kJ/mol (Figure 9D). The 2D interaction structures were visualized using the Zentrum für Bioinformatik website (Figure 10A). Quantitative PCR analysis revealed that EA and EB downregulated FAK1 and FAK2 gene expression in BMMs (Figure 10B) while upregulating their expression in cranial preosteoblasts at very high doses (Figure 10C). These findings suggest that EA and EB may regulate the FAK signaling pathway by interacting with both FAK1 and FAK2, contributing to their effects on osteoclast and osteoblast differentiation.

### EA and EB suppress the activation of the FAK-PI3K signaling pathway in vivo

To investigate the regulatory effects of EA and EB on the FAK-PI3K signaling pathway, we conducted histological and molecular analyses. Trap staining (Supplementary Figure S5A) revealed a significant reduction in osteoclast-positive areas in the EA and EB treatment groups compared to the OVX group, exhibiting a dose-dependent trend. Quantitative analysis of the Trap-positive area ratio (Supplementary Figure S5B) confirmed this trend, suggesting that EA and EB may exert inhibitory effects on osteoclast activity.

To further elucidate the underlying molecular mechanisms, we performed Western blot analysis to assess the protein expression levels of key components of the FAK-PI3K signaling pathway, including FAK (FAK1), PTK2B (FAK2), PI3K, and phosphorylated PI3K (P-PI3K) (Supplementary Figure S5C). Quantitative analysis (Supplementary Figures S5D–G) demonstrated a significant upregulation of FAK and PTK2B expression in the OVX group. Compared to OVX, EA and EB treatments markedly suppressed FAK expression (p < 0.0001) and led to a moderate decrease in PTK2B levels (p < 0.001). Furthermore, the expression levels of PI3K and P-PI3K were significantly reduced following EA and EB treatment, indicating inhibition of the FAK-PI3K signaling pathway activation.

Immunofluorescence staining (Supplementary Figures S5H, I) further corroborated these findings. Compared to the OVX group, the fluorescence intensities of FAK, PTK2B, PI3K, and P-PI3K were markedly decreased in the EA and EB treatment groups. Quantitative analysis (Supplementary Figures S5J–M) confirmed significant reductions in the fluorescence intensities of FAK (p < 0.0001), PTK2B (p < 0.0001), PI3K (p < 0.0001), and P-PI3K (p < 0.0001), providing strong evidence that EA and EB effectively suppress the activation of the FAK-PI3K signaling pathway *in vivo*.

## Discussion

*Epimedium*, a genus within the Berberidaceae family, comprises approximately 52 species (Guo et al., 2022) and contains over 270 distinct compounds (Tong et al., 2023). As a traditional Chinese medicine (Ma et al., 2011), *Epimedium* has demonstrated various pharmacological effects, including antioxidative (Li N. et al., 2019), antimicrobial (Li and Zhang, 2013), anti-osteoporotic (Zhang et al., 2007), and anticancer properties (Zhang et al., 2014), as substantiated by modern pharmacological studies and clinical practice (Li N. et al., 2019). Through innovative network pharmacological analysis, we identified five monomeric compounds from *Epimedium* that are rarely reported in the literature and may possess potential anti-osteoporotic effects (Liu et al., 2021). Consequently, investigating the active compounds of *Epimedium* holds significant promise for the discovery of new anti-osteoporosis drugs and for elucidating novel mechanisms to combat osteoporosis.

Network pharmacology represents a cutting-edge approach in drug discovery, especially in the context of the current era characterized by artificial intelligence and big data (Zhang et al., 2023). This methodology seeks to explore the core interactions within biomolecular networks related to diseases and syndromes, thereby identifying key pharmacodynamic components in Chinese medicine formulations and elucidating their mechanisms of action in treating various conditions (Li L. et al., 2019). Network pharmacology not only provides robust data to support the rational clinical use of drugs but also facilitates the development of new drugs within the domain of Chinese medicine (Zhao et al., 2023). However, current research has predominantly focused on clinical trials or pharmacodynamic studies, with limited exploration of the underlying mechanisms by which Chinese medicine formulations exert their therapeutic effects in osteoporosis, particularly through network pharmacology (Li et al., 2023). Therefore, in our study, we adopted an integrative approach combining network pharmacology and molecular docking to investigate osteoporosis, laying a foundation for uncovering its underlying mechanisms.

Based on preliminary research, differences have been observed between the active ingredients of traditional Chinese medicine prescriptions and those of single-component Chinese medicines (Shen et al., 2017). When utilizing research data platforms, considerations such as oral bioavailability and drug-likeness during the screening process for effective components in Chinese medicine may lead to the inadvertent omission of many active molecules. Thus, our study initially focuses on identifying the primary components of *Epimedium* using liquid chromatography-mass spectrometry (LC-MS) (Wang et al., 2023). Subsequently, leveraging chemical analysis results, we employed network pharmacology, molecular docking, and molecular dynamics simulations to elucidate the mechanisms by which *Epimedium* monomeric compounds exert their anti-osteoporotic effects.

Our investigation revealed that EA and EB inhibit osteoclast differentiation while promoting osteoblast differentiation, findings that are consistent with previous studies. We utilized five open-access resources to predict the targets of EA and EB, followed by Ingenuity Pathway Analysis (IPA) databases to predict the signaling pathways regulated by these compounds (Young et al., 2009; Ma et al., 2023). Although IPA provides pre-built analysis pipelines, which may limit customization of analysis parameters or the incorporation of user-defined pathways, it nonetheless integrates various omics data types to enable a comprehensive analysis of biological systems (Liu et al., 2022). The prediction of EA and EB target pathways identified the FAK signaling pathway as a key regulatory mechanism. Previous studies have underscored the critical role of PYK2/FAK kinases in maintaining bone homeostasis, particularly in the processes of bone formation and resorption. FAK1 and PYK2 are key proteins in the FAK signaling pathway. Previous studies have demonstrated that PYK2/FAK kinases play a crucial role in maintaining bone homeostasis. During bone formation, FAK is continuously activated throughout the osteogenic differentiation of human mesenchymal stem cells (hMSCs) (Liao et al., 2013). When hMSCs are stimulated to differentiate into osteoblasts, FAK activity is enhanced (Liao et al., 2012). Knockdown of FAK significantly reduces the activation of the Wnt/β-catenin and MAPK signaling pathways, and effectively inhibits BMP-9-induced bone formation *in vivo* (Zheng et al., 2019). Using siRNA against FAK (siFAK) or in FAK (−/−) osteoblasts to impair FAK activity leads to a reduction in osteopontin (OPN) expression in osteoblasts (Young et al., 2009). Regarding bone resorption, Ray et al. found that the FAK family is involved in regulating osteoclast structure and function by establishing transgenic mice in which FAK was selectively deleted in osteoclast precursors (Ray et al., 2012). However, the role of FAK in osteoclasts remains relatively unexplored, presenting a novel therapeutic target for osteoporosis.

Our study highlights the therapeutic potential of EA and EB in treating osteoporosis, supported by *in vivo* experiments demonstrating their efficacy. To further elucidate the mechanisms of action of EA and EB in osteoporosis, we employed IPA and molecular docking techniques to predict target interactions. The results indicated that EA and EB interact with the FAK protein, thereby regulating the FAK signaling pathway. These findings suggest that EA and EB may serve as novel therapeutic agents for osteoporosis. Understanding their mechanisms of action in the pathogenesis of osteoporosis, particularly their effects on osteoblast and osteoclast activity, will be crucial and warrants further investigation.

## Data Availability

The original contributions presented in the study are included in the article/Supplementary Material, further inquiries can be directed to the corresponding authors.

## Abbreviations

OP, Osteoporosis; BMD, Bone Mineral Density; OVX, Ovariectomy; ODM, Osteoblastogenesis Experiments; EA, Epimedin A; EB, Epimedin B; EPA, Epimedoside A; PHBA, 4-Hydroxybenzaldehyde; GA, Genetic Algorithm; BV/TV, Bone Volume/Tissue Volume; Tb.Th, Trabecular Thickness; Tb.N, Trabecular Number; Conn.Dn, Connection Density; BS, Bone Surface; Tb.Sp, Trabecular Separation; BMMs, Bone Marrow-Derived Macrophages/Monocytes; ALP, Alkaline Phosphatase.
