# Supplementary material for: Integrating network pharmacology, IPA, and molecular docking to reveal the anti-osteoporosis effects of EA and EB via the FAK pathway
Source: Front Pharmacol. 2025 Jul 2;16:1532665. doi: 10.3389/fphar.2025.1532665 (PMC12264438; doi:10.3389/fphar.2025.1532665)
Supplement: Supplementary file 1 [file DataSheet1.zip › Supplementary Material.docx]

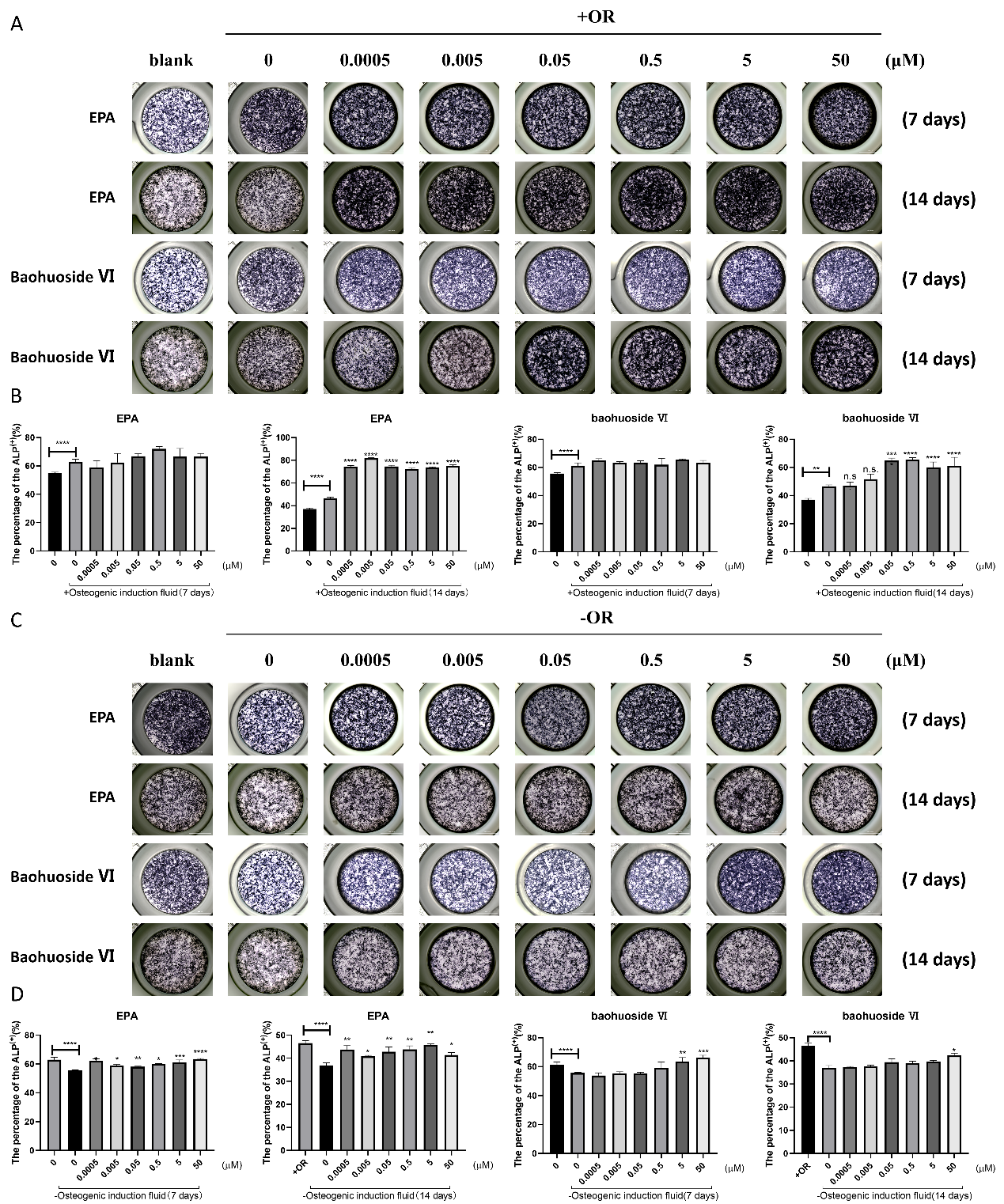


**Supplementary Figure 1. EPA and Baohuoside VI promote osteoblast formation without cytotoxicity in vitro.**
A. Cranial osteoblasts were stimulated with (A) and without (C) osteogenic induction medium (OR) in the presence of varying concentrations of EPA (0, 0.0005, 0.005, 0.05, 0.5, 5, 50 μM) and Baohuoside VI (0, 0.0005, 0.005, 0.05, 0.5, 5, 50 μM). ALP staining was performed on days 7 and 14.
B, D. Quantitative analysis of ALP-positive cells. *P<0.05; **P<0.01; ***P<0.001; ****P<0.0001 compared to the control group.


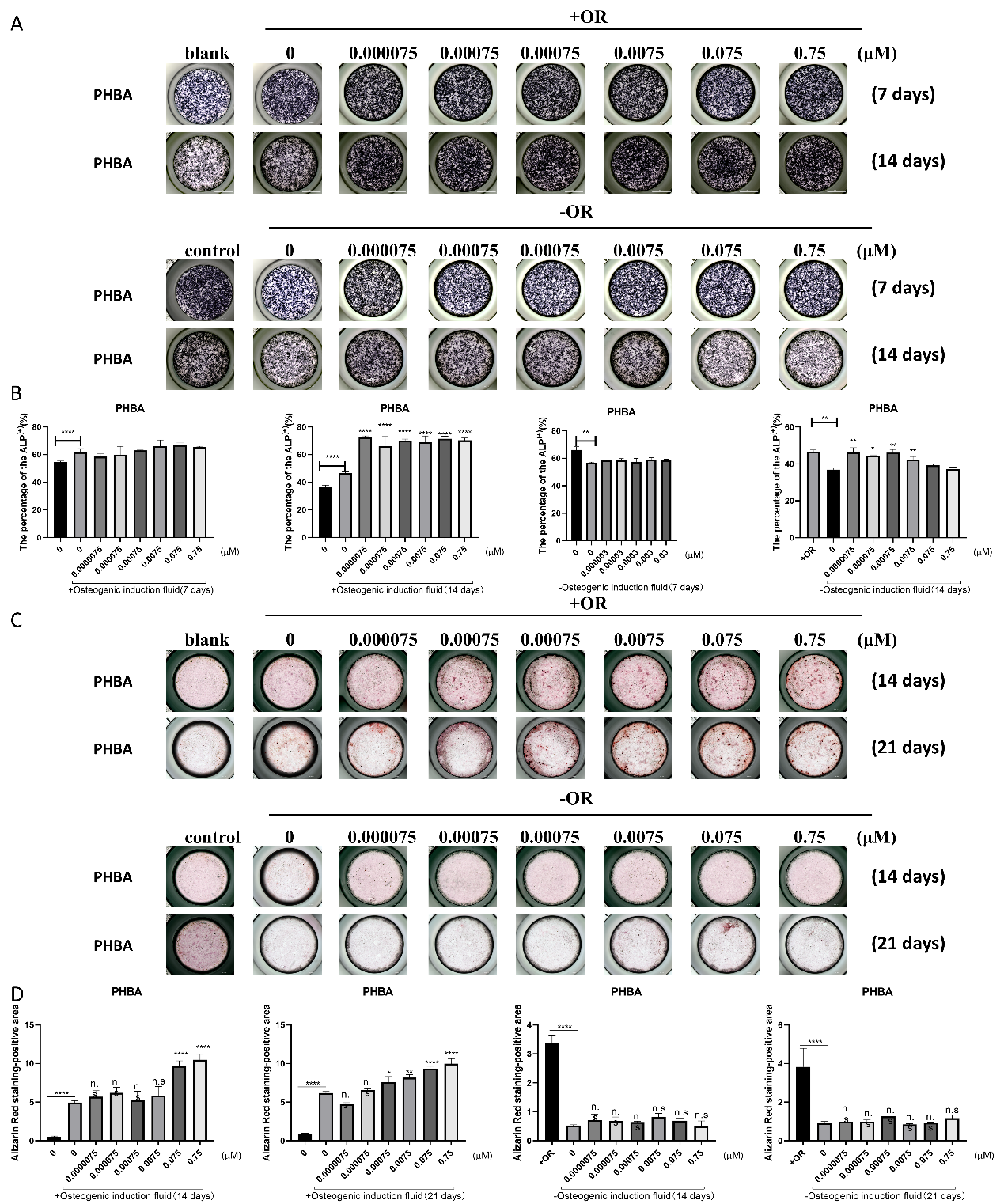


**Supplementary Figure 2. PHBA promotes osteoblast formation without cytotoxicity in vitro.**
A. Cranial osteoblasts were stimulated with and without osteogenic induction medium (OR) in the presence of varying concentrations of PHBA (0, 0.0000075, 0.000075, 0.00075, 0.0075, 0.075, 0.75 μM) and stained for ALP detection on days 7 and 14.
C. Cranial osteoblasts were stimulated with and without OR in the presence of varying concentrations of PHBA (0, 0.0000075, 0.000075, 0.00075, 0.0075, 0.075, 0.75 μM) and stained for Alizarin Red detection on days 14 and 21.
B. Quantitative analysis of ALP-positive cells.
D. Quantitative analysis of Alizarin Red staining-positive cells. *P<0.05; **P<0.01; ***P<0.001; ****P<0.0001 compared to the control group.


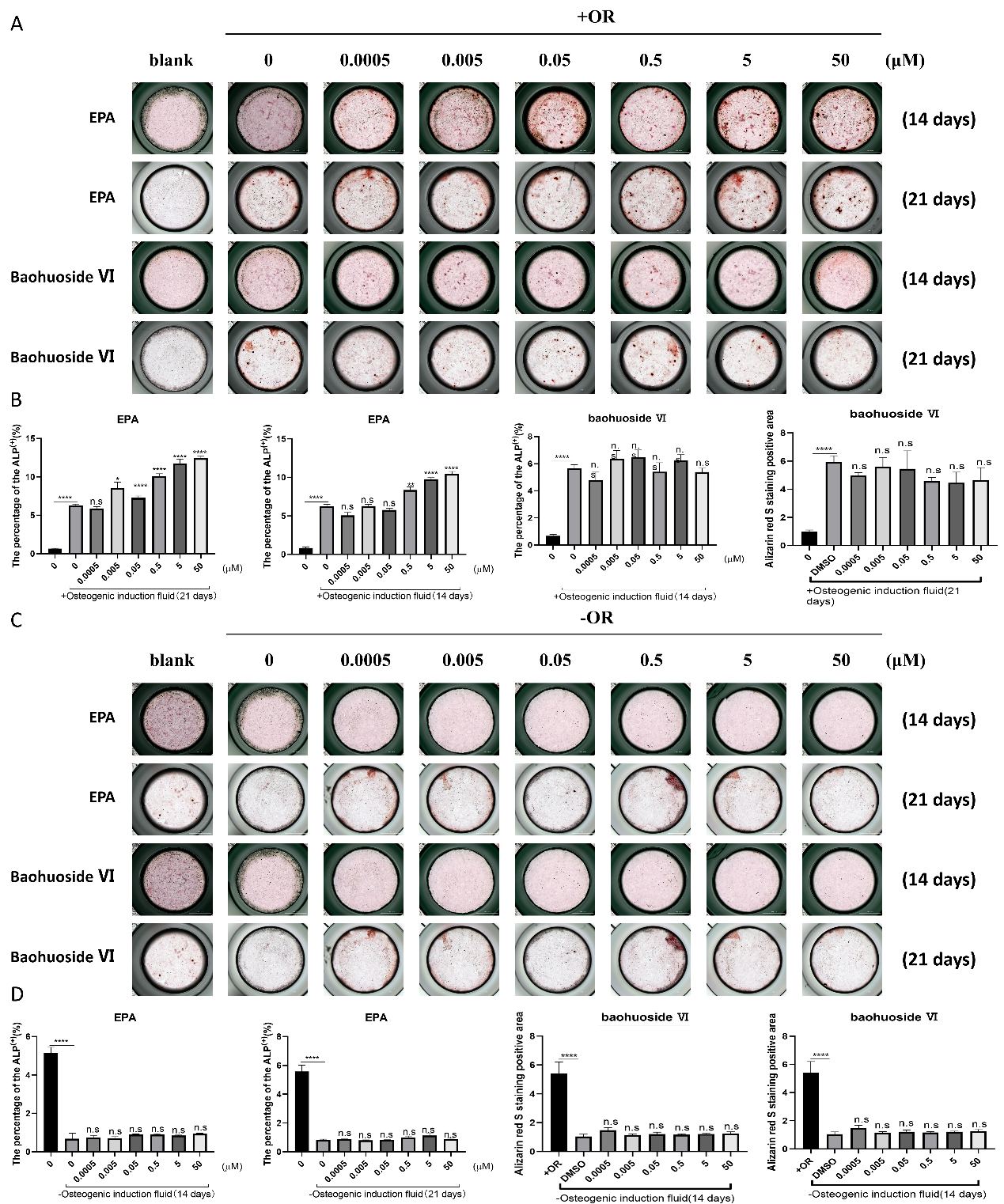


**Supplementary Figure 3. EPA and Baohuoside VI enhance calcium deposition in osteoblasts in vitro.**
A. Cranial osteoblasts were stimulated with (A) and without (C) osteogenic induction medium (OR) in the presence of varying concentrations of EPA (0, 0.0005, 0.005, 0.05, 0.5, 5, 50 μM) and Baohuoside VI (0, 0.0005, 0.005, 0.05, 0.5, 5, 50 μM). Alizarin Red staining was performed on days 14 and 21.
B, D. Quantitative analysis of Alizarin Red staining-positive cells. *P<0.05; **P<0.01; ***P<0.001; ****P<0.0001 compared to the control group.


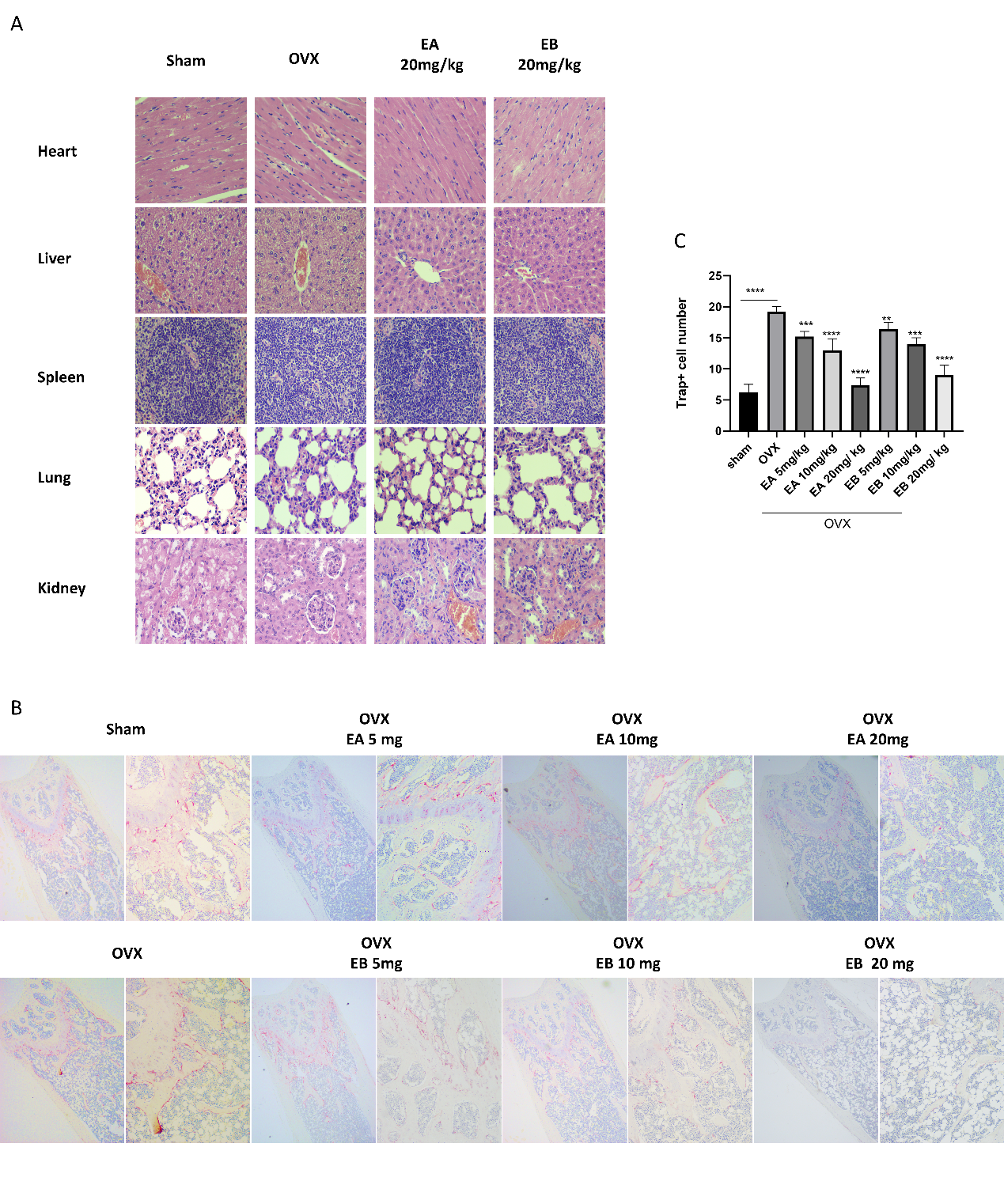


**Supplementary Figure 4. H&E and TRAP staining from the animal experiment.**
A. Representative images of H&E staining for the heart, liver, spleen, lung, and kidney tissues from each group, used to evaluate the toxicity of EA and EB.
B. Representative images of TRAP staining for each group.
C. The number of TRAP-positive multinucleated osteoclasts on the trabecular bone surface is presented in the bar chart. All bar graphs show the mean ± SD. **P<0.01; ***P<0.001; ****P<0.0001 compared to the OVX group.


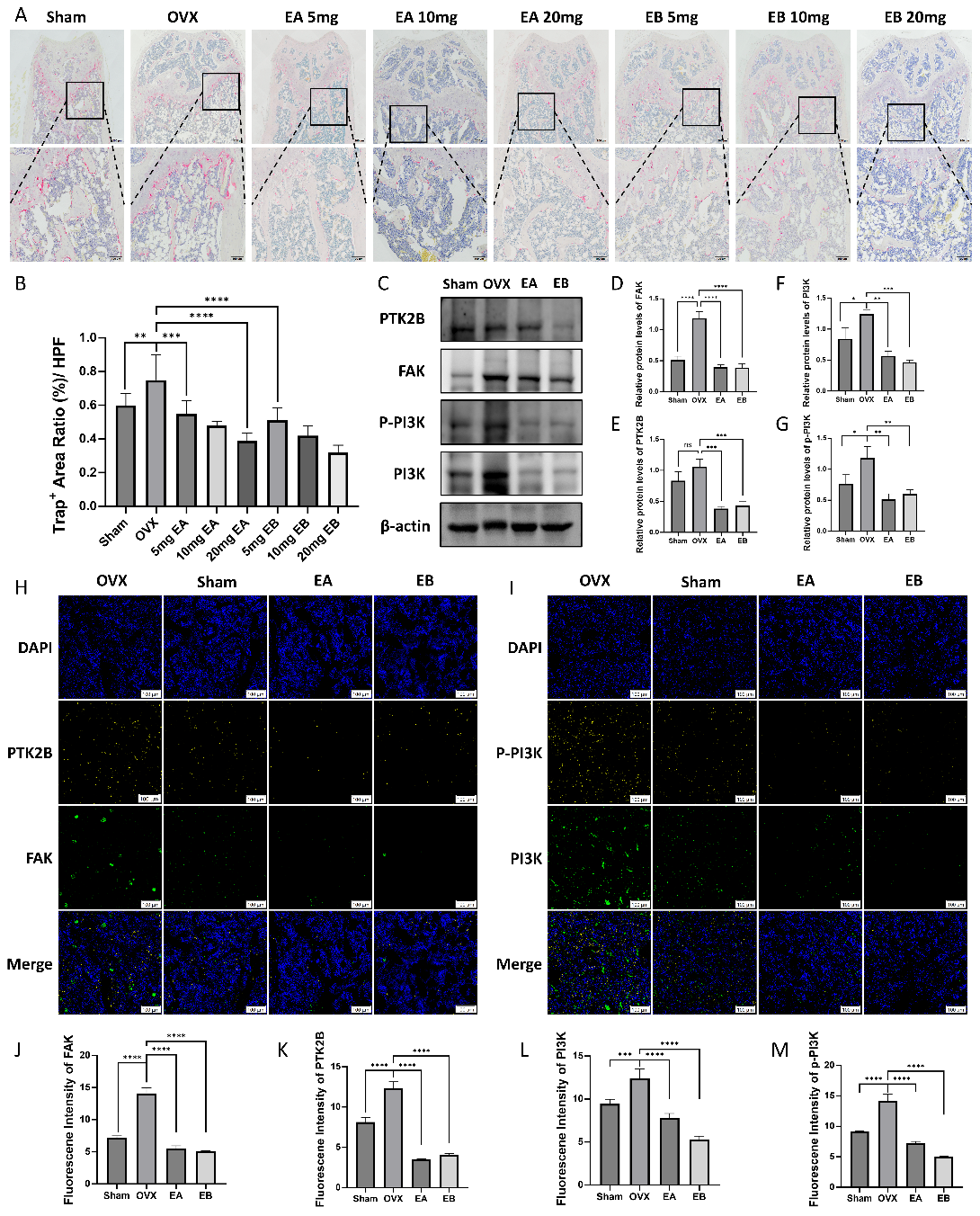


**Supplement Figure 5. The effect of EA and EB on the FAK signaling pathway.**
A. Representative images of Trap-stained sections from each treatment group.

B. Quantitative analysis of the ratio of Trap-positive areas.

C. The effects of EA and EB on the FAK signaling pathway were verified by Western Blot using specific antibodies against FAK, PTK2B, PI3K and P-PI3K. β-actin served as the loading control.

D-G. Quantitative analysis of the relative protein level of FAK, PTK2B, PI3K and P-PI3K.

H. Immunofluorescent staining to evaluate the expression of FAK (green), PTK2B/FAK2 (yellow) and nuclei (blue).

I. Immunofluorescent staining to evaluate the expression of PI3K (green), P-PI3K (yellow) and nuclei (blue).

J-M. Quantitative analysis of the fluorescence intensity of FAK, PTK2B, PI3K and P-PI3K in (H, I). Results are presented as mean ± SD. Statistical significance was tested by one-way ANOVA with Tukey’s multiple comparison test. (ns, no significant difference; *p < 0.05; **p < 0.01; ***p < 0.001; ****p < 0.0001).

**Supplementary File 1. Target prediction for EA and EB.**
Target predictions for EA and EB were performed using four online resources, and the interaction results were used in subsequent analyses.

**Supplementary File 2. Secondary bonds formed by ligand-protein binding.**
Details of the secondary bonds in the binding pocket, including hydrogen bonds, hydrophobic bonds, and π-bonds, with the required binding free energy across 50 operations.

**Supplementary File 3. Interaction energy between ligand and protein.**
A comprehensive list of all results showing binding energies below < −1.2 kcal/mol or < −5 kJ/mol for 50 operations.

**Supplementary Table 1. Database of Anti-Osteoporosis Active Ingredients in *Epimedium***
